# The Relationship Between Blood Sample Volume and Diagnostic Sensitivity of Blood Culture for Typhoid and Paratyphoid Fever: A Systematic Review and Meta-Analysis

**DOI:** 10.1093/infdis/jiy471

**Published:** 2018-10-11

**Authors:** Marina Antillon, Neil J Saad, Stephen Baker, Andrew J Pollard, Virginia E Pitzer

**Affiliations:** 1 Department of Epidemiology of Microbial Diseases, Yale School of Public Health, New Haven, Connecticut; 2 Center for Health Economics Research and Modeling of Infectious Diseases, University of Antwerp, Belgium; 3 Oxford University Clinical Research Unit Vietnam, Ho Chi Minh City, Vietnam; 4 Department of Paediatrics, University of Oxford , Oxford, United Kingdom

**Keywords:** blood culture, bone marrow culture, diagnostic accuracy, paratyphoid fever, typhoid fever

## Abstract

**Background:**

Blood culture is the standard diagnostic method for typhoid and paratyphoid (enteric) fever in surveillance studies and clinical trials, but sensitivity is widely acknowledged to be suboptimal. We conducted a systematic review and meta-analysis to examine sources of heterogeneity across studies and quantified the effect of blood volume.

**Methods:**

We searched the literature to identify all studies that performed blood culture alongside bone marrow culture (a gold standard) to detect cases of enteric fever. We performed a meta-regression analysis to quantify the relationship between blood sample volume and diagnostic sensitivity. Furthermore, we evaluated the impact of patient age, antimicrobial use, and symptom duration on sensitivity.

**Results:**

We estimated blood culture diagnostic sensitivity was 0.59 (95% confidence interval [CI], 0.54–0.64) with significant between-study heterogeneity (*I*^*2*^, 76% [95% CI, 68%–82%]; *P* < .01). Sensitivity ranged from 0.51 (95% CI, 0.44–0.57) for a 2-mL blood specimen to 0.65 (95% CI, 0.58–0.70) for a 10-mL blood specimen, indicative of a relationship between specimen volume and sensitivity. Subgroup analysis showed significant heterogeneity by patient age and a weak trend towards higher sensitivity among more recent studies. Sensitivity was 34% lower (95% CI, 4%–54%) among patients with prior antimicrobial use and 31% lower after the first week of symptoms (95% CI, 19%–41%). There was no evidence of confounding by patient age, antimicrobial use, symptom duration, or study date on the relationship between specimen volume and sensitivity.

**Conclusions:**

The relationship between the blood sample volume and culture sensitivity should be accounted for in incidence and next-generation diagnostic studies.

*Salmonella enterica* serovar Typhi is the causative agent of typhoid fever, for which there are an estimated 17.8 million cases per year; *S enterica* serovars Paratyphi A, B, and C cause a clinically indistinguishable syndrome and contribute an additional 4.6 million cases per year [1, 2]. Most of this burden occurs in low- and middle-income countries (LMICs), where the clinical presentation of typhoid fever overlaps with the symptoms of many other diseases, such as malaria and dengue [3].

For a definitive diagnosis of typhoid or paratyphoid (enteric) fever, the World Health Organization recommends bacterial isolation from blood or bone marrow [4]. Bone marrow culture (the gold standard) is acquired via aspirate of the iliac crest or sternum and has a suggested sensitivity of ~90% after 4 days of culture [5, 6]. However, due to the invasive nature of bone marrow biopsies, the diagnosis of enteric fever in LMICs typically depends on blood culture or the Widal test, an antibody titer test with markedly poor specificity (~77%) [4, 7, 8]. Blood culture has been the de facto diagnostic test for population-based incidence studies, vaccine trials, as well as the reference standard for novel diagnostics [6, 9, 10].

Blood culture diagnostic sensitivity is characterized by heterogeneity, with estimates ranging between 40% and 87% [6]. Some of this variation could be explained by blood sample volume—a relationship that is commonly acknowledged but has never been quantified [6, 11–14]. Specimen volume varies markedly between studies depending on local regulations and/or the patient’s age [9, 15–17]. Moreover, factors such as patient age (independent of specimen volume), symptom duration, previous antimicrobial use, and laboratory culture conditions (broth and agar used, automated culture systems) are hypothesized to influence blood culture sensitivity [6, 13, 18–22].

We conducted a systematic review and meta-analysis to explore how patient attributes and specimen volume contribute to heterogeneity in estimates of blood culture sensitivity. We investigated how the relationship between blood volume and culture sensitivity could be impacted by the age of the patient, the duration of disease before specimen collection, and prior antimicrobial use.

## MATERIALS AND METHODS

We conducted our systematic review according to an a priori-specified protocol (see, Supplement S1 & S2) and adhered to the Preferred Reporting Items for Systematic Reviews and Meta-Analyses (PRISMA) guidelines for reporting [23]. One author (M.A.) performed the search and screening. The data extraction and risk of bias assessment were conducted in duplicate (M.A. and N.J.S.), and discrepancies were resolved through consensus or by a third reviewer (V.E.P.).

### Inclusion Criteria and Search Strategy

We searched Web of Science, MEDLINE, Embase, Global Health, and PubMed Central for studies published before June 19, 2016, without additional date or language restriction. We identified relevant publications using controlled vocabulary and free text related to enteric fever and blood or bone marrow culture (see Supplement S1). We defined eligible publications as epidemiological studies of any design that assessed the sensitivity of blood culture to detect typhoid or paratyphoid among patients who also had at least 1 sample of blood and bone marrow cultured for either infection. We did not include editorials, commentaries, or reviews (see Supplement S2). We screened the title and abstract of the studies identified, confirmed eligibility by full-text review, and identified additional articles by cross-checking the references of original articles and reviews.

### Data Extraction

We extracted data on the number of positive and negative blood and bone marrow cultures, as well as the volume of each sample, if reported (see Supplement S3). We also obtained data on the number of additional patients identified by positive cultures of other sites (rose spots, rectal swab, stool, urine). Because some studies did not report the concordance between blood and bone marrow culture results, we calculated the proportion of blood cultures that grew colonies of *S* Typhi or *S* Paratyphi (A, B, or C) among individuals with at least 1 positive culture from any site, thereby leveraging as many studies as possible.

### Risk of Bias Assessment

We modified the QUADAS-II tool [24] to fit our research question and assessed the risk of bias in patient recruitment, index test (blood culture), reference test (bone marrow culture), or the flow and timing of the 2 cultures (see Supplement S3).

### Statistical Analysis

We used an inverse-variance weighted binomial-normal model to estimate the overall diagnostic sensitivity of blood cultures of *S* Typhi or *S* Paratyphi and used a random effects model to account for unmeasured heterogeneity between studies, which was quantified using the *I*^*2*^ statistic [25].

To investigate factors that contribute to heterogeneity of blood culture sensitivity estimates across studies, we performed a meta-regression analysis to assess the relationship between blood specimen volume and sensitivity. We specified the relationship between blood culture sensitivity and specimen volume in 3 ways. First, we tested a “null” model in which diagnostic sensitivity is independent of specimen volume. A second and third model assumed that sensitivity had a log-linear relationship with volume using a slope-only and a slope-and-intercept functional form, respectively (see Supplement S4, section 4.1). We compared the models based on Widely Applicable Information Criterion (WAIC) [26]. We also modeled sensitivity using a linear meta-regression model to assess the impact of the functional form on the relationship between specimen volume and sensitivity (see Supplement S4, section 4.1). We verified our results were robust to the definition of “true positives” (positive culture from any site) by re-estimating blood culture sensitivity and its relationship with specimen volume among patients who were specifically bone marrow culture-positive; studies that did not report the number of patients who were blood culture-positive strictly among those who were also positive by bone marrow culture were excluded from this analysis (n = 3).

Next, we performed a subgroup analysis to test whether heterogeneity across studies was attributable to the differences in the age of patients in the study. To evaluate whether age had a confounding or modifying effect on the relationship between specimen volume and blood culture sensitivity, we included age as an independent predictor and interaction term in the primary meta-regression model (see Supplement S4, section 4.2).

We also performed a subgroup analysis on the sensitivity reported in studies according to the date of publication (before 1980, 1980–1990, and after 1990, which partitions the studies into 3 approximately equal groups). Moreover, we performed meta-analyses on the diagnostic risk ratio of the duration of symptoms and prior antibiotic use, and we tested for a modifying effect of specimen volume on these risk ratios (see Supplement S4, section 4.3).

Analyses were performed in R version 3.3.2 and JAGS version 3.4.0 (details are found in Supplement S4, section 4.4).

## RESULTS

### Inclusion and Characteristics of Studies

**Figure 1.**
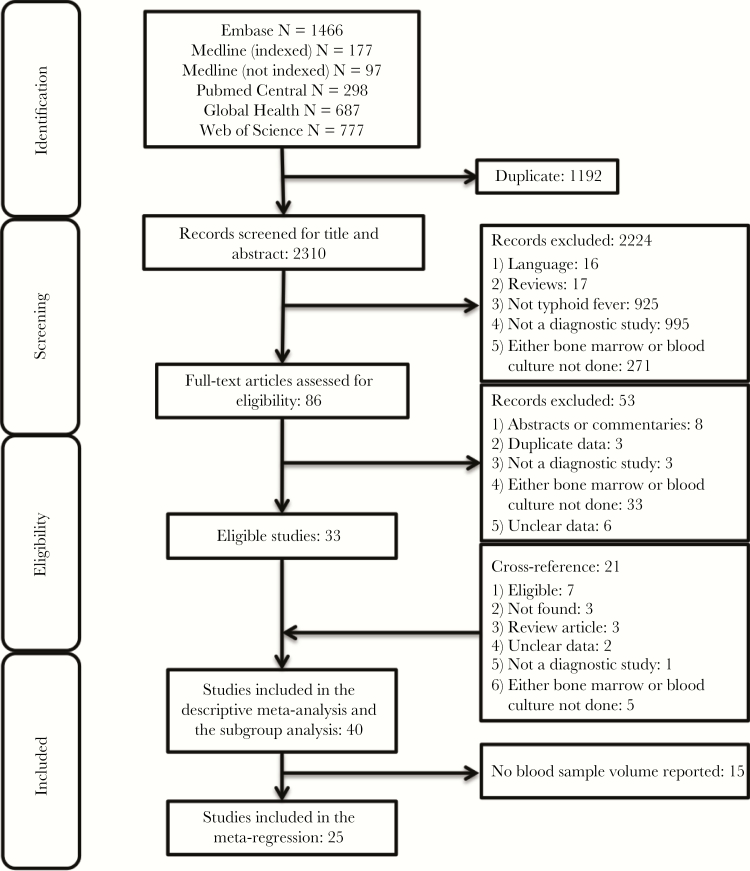
Preferred Reporting Items for Systematic Reviews and Meta-Analyses (PRISMA) diagram for systematic review. A systematic search in 6 databases yielded 3502 articles, or 2310 unique articles, 86 of which were eligible for full-text review. A total of 40 studies were included in the descriptive synthesis, and 25 studies had blood volume information and were included in our meta-regression analysis of the relationship between blood sample volume and blood culture sensitivity.

After screening the titles and abstracts of 2310 unique articles identified in our search, 86 articles were identified for full-text screening, yielding a preliminary set of 33 studies for data extraction. After cross-checking the reference lists of the preliminary set and of systematic reviews, we identified an additional 21 articles for full-text review, of which 7 additional studies were eligible (Figure 1). In total, 40 studies were included in the systematic review and meta-analysis, yielding a total of 2051 patients; 25 studies reported blood specimen volume, yielding 1370 patients for the meta-regression analysis. Reasons for exclusion are reported in Figure 1.

### Characteristics of the Studies Included

Table 1 presents the characteristics of the included studies; additional details are provided in Supplementary Table S1. All but 4 studies (10%) were conducted in LMICs, and 38 studies (95%) were conducted in urban areas. In most studies, eligibility criteria consisted of “clinical suspicion” of enteric fever (which was rarely described), but in 2 studies (Supplemental Material, Bassily 1980 and Bhutta 1991), the patient populations were individuals suspected of chronic carriage of typhoid or individuals who had prolonged symptoms, respectively. Blood cultures were performed using a variety of techniques (Supplementary Table 1); selective media were generally used, and a few more recent studies have used techniques to improve blood culture sensitivity, such as cell lysis and centrifugation; only 1 study reported using a Bactec (automated) system (see Supplementary Table S1, Escamilla 1982). Three studies (7.5%) were conducted among patients of all ages, 21 studies (52.5%) were performed in a subset of age groups, and 16 studies (40%) did not specify patient age. Seven studies (17.5%) reported sensitivity stratified by antimicrobial use, and 15 studies (37.5%) reported sensitivity according to the time from symptom onset to sample collection.

**Table 1.** Study Characteristics^a^

Study (Language, if Other Than English)	Location	Typhi, Paratyphi Included	Eligibility Criteria	Inpatients or Outpatients	Age Distribution of the Sample (in Years)^c^	How Long the Blood Was Incubated	Broth Used to Culture the Blood	Agar Used to Subculture the Blood	Other Cultures
Akoh 1991 [13]	Zaria, Nigeria	Typhi	Clinical suspicion	Inpatient	Not reported	Not reported	Thioglycolate	Not reported	Urine and stool
Avendano 1986 [14]	Santiago, Chile	Typhi, Paratyphi	Clinical suspicion	Inpatient	Children (ages 3–14)	Not reported	Brain heart infusion with sodium polyanetholsulfonate	*Salmonella*-*Shigella*, bismuth sulfite, Kliglers triple sugar iron	Bile
Baqi Durrani 1996 [15]	Karachi, Pakistan	Typhi, Paratyphi	Clinical suspicion or 4× rise in titers	Both	Older children (ages 5–20)	7 days	Thioglycolate	Sheep’s blood, MacConkey’s, *Salmonella*-*Shigella* agar plates	
Barbagallo 1938 (Italian) [16]	Catania, Italy	Typhi, Paratyphi	Clinical suspicion	Inpatient	Not reported	Not reported	Oxoid (trypticase-soy broth)	MacConkey’s, triple sugar iron	
Bassily 1980^a^[17]	Cairo, Egypt	Typhi, Paratyphi	Patients suspected to have chronic salmonellosis	Inpatient	Older children (ages 10–18)	Not reported	Oxgall (oxbile)	Not reported	Urine culture, data not shown
Benavente 1981 [18]	Lima, Peru	Unclear or not reported	Not reported	Both	Not reported	Not reported	Oxgall (oxbile)	Not reported	Stool and bile cultures
Benavente 1984 [19]	Lima, Peru	Typhi	Clinical suspicion	Unclear or not reported	Not reported	2+ days	Oxgall (oxbile)	*Salmonella*-*Shigella*	Stool cultures, duodenal cultures
Bhutta 1991^a^ [20]	Karachi, Pakistan	Typhi	Patients with prior antibiotic treatment or a long duration of illness	Unclear or not reported	Children (age range not reported)	Not reported	Not reported	Not reported	
Chaicumpa 1992 [21]	Jakarta, Indonesia	Typhi	Clinical diagnosis	Inpatient	Not reported	7 days	Oxgall (oxbile)	MacConkey’s, *Salmonella*-*Shigella*, desoxycholate-citrate-sucrose-lactose	Urine and stool
Chang 1982 (Spanish)^a^ [22]	Lima, Peru	Typhi	Agglutination >1/160 or “significant” rise in titers, or a positive culture	Unclear or not reported	Children (age range not reported)	Not reported	Not reported	Not reported	
Chiragh 2005^a^ [23]	Peshawar, Pakistan	Typhi	Clinical suspicion and a fever of more than 4–5 days; no antibiotics taken in previous 3–4 days	Unclear or not reported	Adults (ages 15+)	Not reported	Not reported	Not reported	Urine, data not shown
Dance 1991 [24]	Kathmandu, Nepal	Typhi, Paratyphi	Clinical diagnosis	Unclear or not reported	Not reported	Not reported	Brain heart infusion containing liquid	Not reported	
Debre 1935 (French)^a^ [25]	Paris, France	Typhi	Not reported	Unclear or not reported	Not reported	Not reported	Meat liver or bile agar	Not reported	
Del Negro 1960 (Portuguese)^a^ [26]	Sao Paulo, Brazil	Typhi, Paratyphi	Culture or serological confirmation of disease	Inpatient	Children (ages 0–15)	Not reported	Not reported	Not reported	Bile, urine, and stool
Farooqui 1991 [27]	Karachi, Pakistan	Typhi	Patients at Aga Khan hospital who had fever of unknown origin	Unclear or not reported	Not reported	7 days	Brain heart infusion and thioglycolate	MacConkey’s, blood	
Gasem 1995 [28]	Semarang, Indonesia	Typhi	Fever for 6+ days and symptoms of typhoid fever	Inpatient	Adults (ages 14–60; mean 23.1)	7 days	Oxgall (oxbile)	*Salmonella Shigella*, triple sugar iron	
Gasem 2003 [29]	Semarang, Indonesia	Typhi	Participants of an RCT of antibiotic treatment of typhoid. Pregnant women were excluded	Inpatient	Adults (ages 14+; mean 24.6; SD 7.7 years)	Not reported	Not reported	Bactec 9120	
Gilman 1975 [30]	Mexico City, Mexico	Typhi	Patients with a clinical suspicion of typhoid who consented to a clinical trial of antibiotics	Unclear or not reported	Not reported	Not reported	Peptone	Not reported	Urine, stool and rose spot cultures
Guerra-Caceres 1979 [31]	Lima, Peru	Typhi	Clinical suspicion	Inpatient	All ages (mean 16.8; median 14; range 2–54)	10 days	Oxoid (trypticase-soy broth) for half the specimens, and Ruiz-Castaneda for the other half	Not reported	Urine and stool
Hirsowitz 1951^b^ [32]	Evaton, Transvaal, South Africa	Unclear or not reported	Culture or serological evidence of typhoid fever, or post-mortem examination consistent with typhoid fever	Inpatient	Older children and adults (ages 10+)	Not reported	Oxgall (oxbile) and nutrient	Not reported	Urine and stool
Hoffman 1984 [10]	Jakarta, Indonesia	Typhi, Paratyphi	Clinical suspicion	Inpatient	Older children and adults (ages 4–60; mean 22; SD 10.3)	7 days	Oxgall (oxbile)	MacConkey’s, *Salmonella*-*Shigella*, desoxycholate-citrate-sucrose-lactose	Bile culture, rose-spot culture
Hoffman 1986 [33]	Jakarta, Indonesia	Typhi, Paratyphi	Clinical suspicion	Inpatient	All ages (mean 21.6; SD 9.4)	21 days	Oxgall (oxbile)	MacConkey’s, *Salmonella*-*Shigella*, desoxycholate-citrate-sucrose-lactose	Streptokinase clot culture and rectal swab
James 1997^b^ [34]	Pondicherry, India	Typhi, Paratyphi	Clinical suspicion and no evidence of chloramphenicol or bone marrow depressant use at the time of specimen collection	Unclear or not reported	Older children and adults (ages 13–42; mean 22.5)	Not reported	Not reported	Not reported	Urine and stool
Ling 1940 [35]	Shanghai, China	Typhi, Paratyphi	Clinical suspicion	Inpatient	Not reported	Not reported	Not reported	Not reported	Urine and stool
Ling 1948 [36]	Shanghai, China	Typhi, Paratyphi	Clinical suspicion	Inpatient	Not reported	7 days	Sodium citrate solution	Endo’s agar	Bile, urine, and stool
Mehta 1984 [37]	Jamnagar, India	Unclear or not reported	Clinical suspicion	Inpatient	Older children and adults (ages 12–40)	2 days	Oxgall (oxbile)	MacConkey’s	
Ott 1938 (German)^b^ [38]	Berlin, Germany	Typhi, Paratyphi	Clinical suspicion	Unclear or not reported	Not reported	Not reported	Oxgall (oxbile)	Not reported	Urine and stool
Rajagopal 1986 [39]	Bangalore, India	Typhi, Paratyphi	Clinical suspicion	Inpatient	Not reported	Not reported	Oxgall (oxbile)	Not reported	Urine and stool
Rubin 1989 [40]	Jakarta, Indonesia	Typhi	Clinical suspicion	Inpatient	Older children and adults (ages 6+)	7 days	Oxgall (oxbile)	MacConkey’s, *Salmonella*-*Shigella*, desoxycholate-citrate-sucrose-lactose	Rectal swabs
Sacks 1941^b^ [41]	Baltimore, MD	Typhi	Clinical suspicion	Inpatient	Older children (ages 11–14)	Not reported	Not reported	Not reported	Stool and urine culture
Schlack 1966 (Spanish) [42]	Santiago, Chile	Typhi, Paratyphi	Clinical suspicion	Inpatient	Children (ages 8 months–13 years)	Unclear	Meat liver broth	Not reported	
Seidenstucker 1949 (German) [43]	Oldenburg, Germany	Typhi, Paratyphi	Not reported	Unclear or not reported	Not reported	2 days	Oxgall (oxbile)	Not reported	Urine and stool
Sekarwana 1989 [44]	Bandung, Indonesia	Typhi	Clinical suspicion and fever of more than 7 days	Unclear or not reported	Children (ages 2–13; mean 6; median 5)	7 days	Oxgall (oxbile)		Urine and stool
Seshadri 1977 [45]	Madras (current-day Chennai), Tamil Nadu, India	Typhi	More than 5 days of fever with toxemia; gastrointestinal symptoms; soft splenomegaly	Unclear or not reported	Older children and adults (ages 13–35; mean 20; median 19)	Unclear	Oxgall (oxbile)	MacConkey’s	Urine and stool
Shin 1994^b^ [46]	Seoul, South Korea	Typhi	Not reported	Unclear or not reported	Adults (ages 20–56)	Not reported	Not reported	Not reported	Urine and stool
Storti 1937 (French)^b^ [47]	Paris, France	Typhi	Clinical suspicion	Inpatient	Not reported	Not reported	Not reported	Not reported	
Terminel 1973 (Spanish)^b^ [48]	Mexico City, Mexico	Typhi	Clinical suspicion among patients who had rose spots	Unclear or not reported	Not reported	Not reported	Not reported	Not reported	Rose spot culture, stool cultures
Vallenas 1985 [49]	Lima, Peru	Typhi	Clinical suspicion	Unclear or not reported	Children (ages 2–13)	Not reported	Oxgall (oxbile)	Not reported	Rectal swab and duodenal culture
Wain 2008 [50]	Ho Chi Minh City, Vietnam and Dong Thap, Vietnam	Typhi	Clinical diagnosis	Inpatient	All ages (age range not reported)	10 days	Oxgall (oxbile), brain heart infusion	Columbia with 0.05% sulphpolyanethosulphonate	Stool, data not shown
West 1989^b^ [51]	Goroka, Papua New Guinea	Typhi	Febrile illness	Inpatient	Older children and adults (ages 10–60; mean 26.3)	21 days	Oxoid (trypticase-soy broth)	Not reported	

Abbreviations: RCT, randomized controlled trial; SD, standard deviation.

^a^Citation numbers correspond to the reference list in Supplement S8. Additional details are provided in Supplementary Table S1.

^b^Indicates that the study will only be included in the summary of the systematic review but not in the analysis examining blood sample volume and sensitivity.

^c^We categorized the age of study populations according to the following criteria: children, 0–4 years; older children, 5–15 years; adults, 15+ years.

### Meta-Analysis

**Figure 2.**
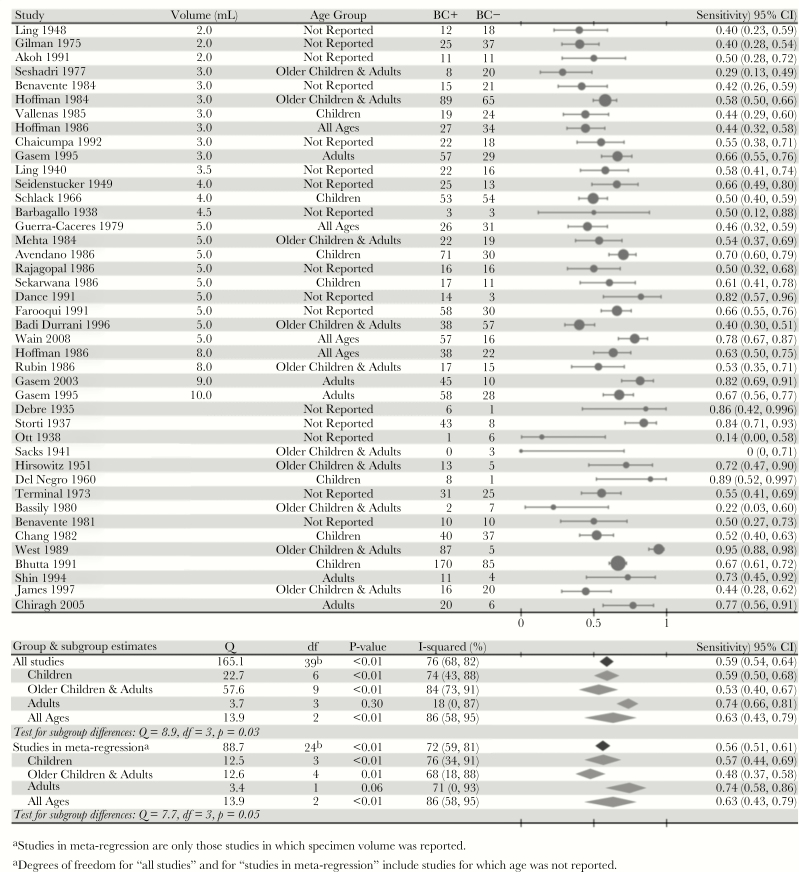
Sensitivity of blood culture to detect typhoid fever. The sensitivity of blood culture is expressed as the proportion of patients who tested positive by blood culture among patients who had at least 1 positive culture (bone marrow, blood, rose spots, stools, or urine) for *Salmonella* Typhi or *Salmonella* Paratyphi. The size of the markers is proportional to the number of patients in the study. We reported the midpoint volume of the blood sample for studies that reported specimen volume as a range. We tested for heterogeneity and age-related subgroup differences via the Q-statistic, which is assumed to have a χ^2^ distribution with degrees of freedom equal to the number of studies minus 1 with noncentrality parameter equal to 0. Hoffman (1986) and Gasem (1995) (Supplemental Material) reported sensitivity on the same patient population using specimens of 2 different volumes per patient, so we have taken only the results from the larger specimen in each study for the subgroup analysis by age to avoid double-counting. Abbreviations: BC+, blood culture-positive; BC−, blood culture-negative; CI, confidence interval.

The overall estimate of blood culture sensitivity was 0.59 (95% CI, 0.54–0.64). Figure 2 shows blood culture sensitivity for each study and subgroup estimates of sensitivity according to the age group of patients. Sensitivity was not markedly different among the subset of studies included in our meta-regression analysis (0.56; 95% CI, 0.51–0.61) or among the 34 studies that reported sensitivity among bone-marrow culture-positive patients only (0.60; 95% CI, 0.53–0.65) (Supplementary Figure S1). However, between-study heterogeneity was statistically significant (*P* < .01) for all of these estimates (Figure 2, Supplementary Figure S1).

#### Specimen Volume

**Figure 3.**
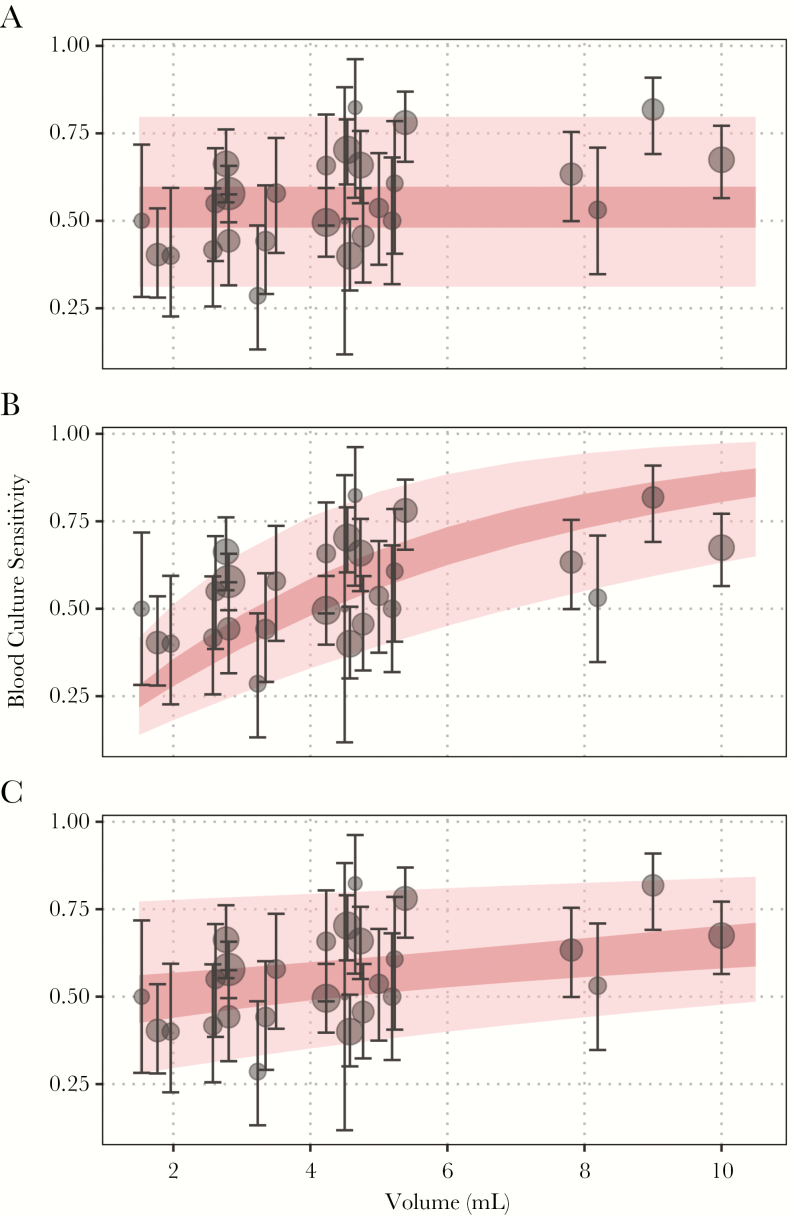
Relationship between sample volume and model estimates of blood culture sensitivity. The observed blood culture sensitivity among all culture-positive cases is plotted in black (with corresponding 95% confidence intervals), whereas the mean model-predicted blood culture sensitivity is plotted in dark pink. The lighter pink regions correspond to the model-predicted population response. (A) The model assumes no correlation with blood volume; (B) the model assumes sensitivity increases with increasing sample volume and is constrained to be zero for a hypothetical 0-mL sample; (C) the model assumes sensitivity could vary with sample volume and estimates an intercept for a hypothetical 0-mL sample. All models account for heterogeneity between studies using random effects (see Supplement S4.1).

We found support for a positive relationship between sample volume and blood culture sensitivity. The observed and model-predicted relationship between sensitivity and sample volume is plotted in Figure 3; model parameters and goodness-of-fit statistics are displayed in Supplementary Table S2. In the preferred model (the model with the lowest WAIC), blood culture sensitivity shows a marginal gain in sensitivity for each additional milliliter of blood and a “baseline” sensitivity for very small samples. The second-best model was one in which sensitivity did not depend on sample volume, whereas the worst fit was obtained for a model that parameterized the relationship between volume and sensitivity using only a log-linear slope term and a fixed (zero) intercept. The results were similar when we modeled blood culture sensitivity among bone-marrow culture-positive patients only, although the baseline sensitivity was higher and the relationship with blood sample volume was attenuated in this analysis (Supplementary Table S2, Supplementary Figure S2). The results were unchanged when we assumed a linear relationship between specimen volume and blood culture sensitivity (Supplementary Table S6).

The mean sensitivity of blood culture was predicted to vary from 0.51 (95% CI, 0.44–0.57) for a 2-mL specimen to 0.65 (95% CI, 0.58–0.70) for a 10-mL specimen (Table 2). Sensitivity increased by 3% (95% CI, 1%–6%) for each additional milliliter of blood cultured (between 1 and 10 mL) according to the linear model (Supplementary Table S6).

**Table 2.** Estimates of Sensitivity by Blood Sample Volume^a^

Volume	Mean Prediction	Population Response
2 mL	0.51 (0.44–0.57)	0.51 (0.30–0.78)
5 mL	0.56 (0.51–0.61)	0.56 (0.38–0.80)
7 mL	0.60 (0.54–0.65)	0.60 (0.42–0.82)
10 mL	0.65 (0.58–0.70)	0.65 (0.46–0.84)

^a^The posterior mean prediction and population response of sensitivity, as well as the corresponding 95% credible intervals, from the log-linear meta-regression model are presented for 2, 5, 7, and 10 mL of blood. The mean prediction is the sensitivity from all studies given a specific volume of blood, whereas the population response is the estimated sensitivity that can be expected from a new study that measures sensitivity with samples of a given volume.

#### Age

Differences between studies could be significantly (*P* = .03) attributed to the differences in age groups recruited. The diagnostic sensitivity was comparable in studies of children (0.59; 95% CI, 0.50–0.68) and in studies that included both older children and adults (0.53; 95% CI, 0.40–0.67), but studies that contained only adults had higher sensitivity (0.74; 95% CI, 0.66–0.81). Between-study heterogeneity within each age group was significant except among the studies that recruited adults only (Figure 2). Results of the subgroup analysis restricted to studies in the meta-regression were comparable, as were the results among patients who were bone marrow culture-positive only (Figure 2, Supplementary Figure S1). When we added age to the best-fit model of specimen volume and sensitivity, the model containing an indicator for age category did not provide a better fit (Supplementary Table S3). There were insufficient data to test for age-related effect modification between specimen volume and sensitivity.

#### Publication Date

There was a subtle trend toward higher sensitivity among studies published after 1990 compared with studies published in 1980–1989 and before 1980 (Supplementary Figure S4), which was only significant among the group of studies included in the meta-regression analysis using data among all culture-positive patients (but not among bone marrow culture-positive patients specifically). There was no evidence that this trend was confounded by patient age or use of improved techniques for culture (Supplementary Table S8). Restricting our analysis of sample volume and sensitivity to studies published after 1980 did not change our choice of best-fit model (Supplementary Table S7, Supplementary Figure S5, and Supplementary Figure S6).

#### Antimicrobial Use and Duration of Illness

**Figure 4.**
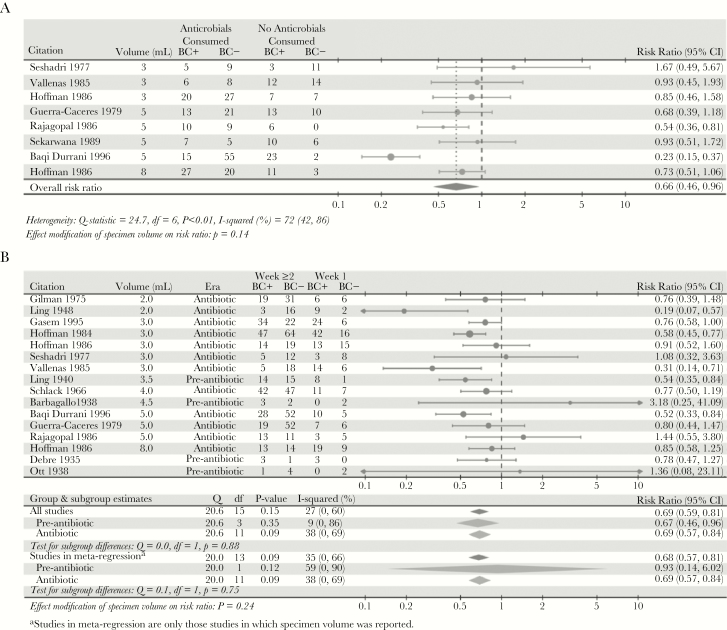
The relative probability of a positive blood culture according to patient history. (A) Relative probability of a positive blood culture for enteric fever patients who took antimicrobials versus patients who did not take antimicrobials before specimen collection. (B) Relative probability of a positive blood culture for enteric fever patients who had blood samples taken for culture in the second week of illness or later versus patients who had blood samples taken for culture in the first week of illness. To assess the possible impact of antibiotics on the relationship between duration of symptoms and sensitivity, we tested for a difference in risk ratio of the duration of illness stratified by studies carried out in the pre-antibiotic era and in the antibiotic era. All studies published before 1945 were considered to report the results of sensitivity in the absence of antimicrobial use, and studies published after 1945 were considered to report results that could show an interaction with antimicrobial use.

Cultures of specimens taken from patients who had been exposed to antimicrobials were 34% (95% CI, 4%–54%) less sensitive than those from patients with no prior antimicrobial use (Figure 4). Furthermore, cultures performed on specimens collected after the first week of illness were 31% (95% CI, 19%–41%) less sensitive than cultures of specimens collected during the first week (Figure 4). We found no modifying effect of antimicrobial use (*P* = .14) or duration of symptoms (*P* = .24) on the relationship between specimen volume and sensitivity. Subgroup analyses showed that the impact of symptom duration on sensitivity was independent of antimicrobial use (*P* = .88) (Figure 4, Supplementary Table S4).

### Risk of Bias Assessment

**Figure 5.**
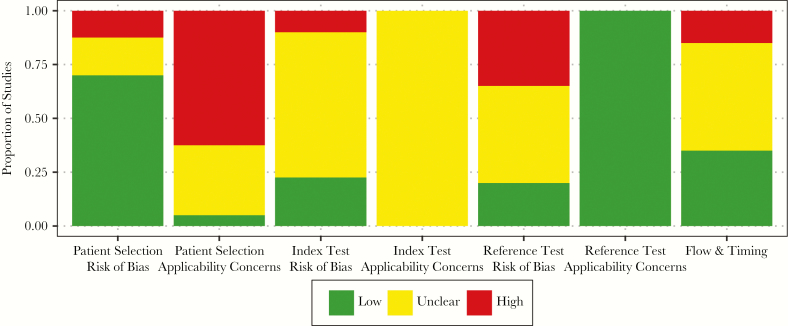
Summary findings of the risk of bias assessment using a modified QUADAS-II tool. We evaluated the risk of bias for the 7 domains of the QUADAS-II tool. The modified QUADAS-II tool was integrated into our data extraction form, found in Supplement S3. Detailed findings on the risk of bias assessment are found in Supplementary Table S6 and discussed in Supplement S5.

The greatest risk of bias involved concerns over the applicability of patient selection (Figure 5, Supplementary Table S5). Twenty-five studies (62.5%) were performed in inpatient (hospitalized) populations and/or in high-income countries. There was also a high risk of bias regarding the reference test (bone marrow culture) in 14 studies (35%) that cultured less than 1 mL of bone marrow (Supplementary Table S1), potentially missing cases with lower bacteremia. For a detailed discussion of the risk of bias assessment, see Supplement S5.

## DISCUSSION

Our systematic review and meta-analysis synthesized the findings of 40 studies and examined factors affecting blood culture diagnostic sensitivity. We identified a significant, although modest, relationship between specimen volume and blood culture sensitivity, resonating with observations that have been made for other infections [27–29], and confirming a relationship that has been postulated but never quantified [6, 11–14]. We estimated that sensitivity of blood culture as a typhoid diagnostic method increases from 51% for 2 mL of blood to 65% for 10 mL, or by 3% for each additional milliliter. Patient age, publication date, antimicrobial use, and duration of symptoms were also associated with changes in blood culture sensitivity, but we found no evidence that these factors confounded or modified the relationship between specimen volume and sensitivity.

Our estimate of the overall sensitivity of blood culture (irrespective of sample volume) is consistent with another recent analysis [14], although the authors of that analysis only identified 10 papers and did not examine the relationship between sensitivity and blood sample volume, antibiotic use, symptom duration, or publication date. Three studies (Supplementary Material; Hoffman 1986, Gasem 1995, Wain 2008) previously examined the relationship between sample volume and blood culture sensitivity directly, but one of these studies did not find conclusive evidence to support the hypothesized relationship (Supplementary Material, Gasem 1995) and another (Supplementary Material, Hoffman 1986) was borderline significant (*P* = .046 by Pearson’s χ^2^ test). We believe these studies were underpowered to detect such an effect. In these 2 studies, between 60 and 86 people were recruited, and the difference in volume between the 2 blood samples collected from each patient was 5–7 mL; according to our analysis, over 400 bone-marrow typhoid-positive patients would need to be recruited to detect a significant difference in sensitivity. In all studies that collected 2 blood samples from the same patient, there was no patient for whom a larger sample of blood failed to confirm diagnosis after isolation of bacteria from the smaller sample; this would be highly unlikely if sensitivity were independent of blood volume (*P* = 3.12 × 10^−36^ assuming 56% sensitivity, as predicted by the meta-analysis) (Figure 2).

To our knowledge, only 1 study has quantified the relationship between blood sample volume and culture sensitivity for other pathogens; it found that each additional milliliter yielded a 3% increase in sensitivity—consistent with our findings—but the relationship could not be established for any one specific pathogen [28]. However, such an effect has been suggested for *Neisseria meningitidis* and *Streptococcus pneumoniae* [30–32]. Only 1 study (which was excluded from this analysis because bone marrow culture was not conducted concurrently) has quantified the effect of age on blood culture sensitivity to detect typhoid [33]. That study found that the density of bacteria is inversely related to age; thus, patient age may confound the relationship between culture volume and sensitivity if smaller samples from children achieve similar sensitivity to larger samples from adults [33, 34]. However, we found the opposite relationship: there was comparable sensitivity in studies consisting of children only and of older children and adults, but higher sensitivity in studies consisting of adults only (Figure 2). Furthermore, there was no evidence of confounding by age when it was included as a predictor in the meta-regression model (Supplementary Table S3).

Sensitivity was also 31% lower among patients whose samples were collected more than 1 week after symptom onset and 34% lower among patients who had been previously exposed to antimicrobials, relationships that had not been assessed in the previous meta-analysis [14]. Similar effects of antimicrobial use on blood culture sensitivity have been observed for *N meningitidis* [28]. The estimated impact of prior antimicrobial use was consistent with the findings from one study in our review (Supplementary Material, Gasem 2003) found that sensitivity decreased by 60% (95% CI, 39%–80%) after 3 days of antimicrobial use and by 73% (95% CI, 50%–89%) after 5 days of antimicrobial use in patients who were blood and bone marrow culture-positive before treatment. Antimicrobial use in that study was defined as a full course of treatment in a setting with limited antimicrobial resistance and completely observed compliance, whereas other studies defined antimicrobial use differently or in vague terms, which may explain the greater effect. Emerging antimicrobial resistance may diminish the negative impact of prior antimicrobial use on blood culture sensitivity.

The relationship between blood specimen volume and diagnostic sensitivity is weak, and considerable uncertainty remains in blood culture sensitivity even after adjusting for specimen volume. Blood cultures may have a low sensitivity for enteric fever because of low bacterial density in the blood, which is often observed in diseases that lack a specific focus of infection [12, 27, 35–37]. In our analysis, only 1 study used an automated culture system (Supplementary Material, Gasem 2003); there was no secular trend in the use of techniques to improve sensitivity (Supplementary Tables S1 and  S8), although there was a weak trend towards higher sensitivity in more modern studies (Supplementary Figure S5). Although the use of automated systems may help to improve culture sensitivity for typhoid, this may not be feasible in many resource-constrained settings where typhoid is endemic; thus, it is also necessary to understand and estimate sensitivity according to the culture methods that are typically used in such settings. Future studies assessing the sensitivity of blood cultures should ideally collect 2 or more blood samples of randomly selected volumes from patients, a sample of bone marrow, and data on age, symptom duration, and antimicrobial use. Our meta-regression framework could then be extended to test the joint effects of patient characteristics on the relationship between specimen volume and sensitivity.

The studies featured in this analysis likely represent a typical typhoid patient population. However, most studies (38 of 40) were exclusively conducted among hospitalized patients, potentially representing more severe cases, whereas only 1%–20% of cases were hospitalized in population-based surveillance studies [16, 38–40]. Moreover, no studies reported the range of symptoms experienced by patients, and, consequently, it remains unclear whether more severely affected patients (potentially with higher bacteremia) were overrepresented in study samples. Furthermore, the sensitivity of bone marrow culture (the gold-standard diagnostic) is itself only ~90%, and <1 mL of bone marrow was collected in 14 studies (Figure 5, Supplementary Table S5). Although we attempted to correct for this by considering patients who were positive according to any specimen as true positives in our primary analysis, some cases may still have been missed, and thus blood culture sensitivity may have been overestimated (Supplementary Figure S8).

The evaluation of next-generation serological diagnostics depends on the appropriate selection of a “gold” (reference) standard to estimate diagnostic accuracy [9, 41–43]. Recent studies of rapid-test diagnostics that used blood cultures as the reference standard collected specimens ranging from 1 to 10 mL [9, 44–46], obfuscating comparisons between studies. Studies that use small blood volumes for culture diagnosis may be selecting for subjects with higher bacteremia, potentially overestimating the sensitivity of the novel diagnostic. Furthermore, such studies may underestimate the specificity of the novel diagnostic test by misclassifying true cases as “false positive” results, especially in high-incidence settings, as shown in a previous modeling study [9, 42]. The data underscores the importance of recruiting typhoid-negative patients from healthy populations or those with a different confirmed infection. Bayesian latent class models have recently been described to account for the imperfect nature of blood culture when evaluating novel diagnostics [44, 47, 48]; our findings can inform prior distributions on the sensitivity of blood culture according to sample volume.

Our findings have important implications for estimates of the burden of typhoid fever and clinical recommendations for the diagnosis of enteric fever. Because the degree of underreporting in surveillance studies depends on the volume of blood collected, meta-regression studies that estimate the global burden of typhoid fever should adjust for differences in culture sensitivity across surveillance studies and between age groups [16, 17, 40]. Finally, wherever ethical and regulatory guidelines permit, more than 7 mL of blood should be drawn for the diagnosis of enteric fever, a recommendation that has been echoed in other hospital-based studies [28].

## CONCLUSIONS

In conclusion, our analysis demonstrates that the sensitivity of blood culture for detecting infection with *S* Typhi and *S* Paratyphi is dependent on sample volume, as well as patient age, duration of symptoms, and prior antimicrobial use. Further prospective studies are needed to better characterize these relationships. The evaluation of novel diagnostics and efforts to integrate data across incidence studies of typhoid fever should take into account the potential influence of these factors.

## Supplementary Data

Supplementary materials are available at *The Journal of Infectious Diseases* online. Consisting of data provided by the authors to benefit the reader, the posted materials are not copyedited and are the sole responsibility of the authors, so questions or comments should be addressed to the corresponding author.

## Supplementary Material

Supplementary Material

## Notes

***Acknowledgments.*** We thank Kate Nyhan at the Yale Medical Library for advice on constructing the systematic review search strategy. We also thank Andrea Torneri for screening and extracting data from Italian publications as well as Katharine Owers and Kathryn Hacker for aiding in screening and extracting data from Portuguese publications.

***Disclaimer.***  The funders had no role in study design, data collection and interpretation, or the decision to submit the work for publication.

***Financial support.*** This work was funded by the Bill and Melinda Gates Foundation (Grants OPP1116967 and OPP1151153; to V. E. P. and M. A.), the Wellcome Trust (Strategic Award 106158/Z/14/Z; to N. J. S., S. B., A. J. P., and V. E. P.), and the Belgian American Educational Foundation Postdoctoral Fellowship program (to N. J. S. and M. A.).

***Supplement sponsorship.*** This article is part of the supplement “Surveillance for Enteric Fever in Asia Project,„ sponsored by the Sabin Vaccine Institute.

***Potential conflicts of interest.*** All authors: No reported conflicts of interest. All authors have submitted the ICMJE Form for Disclosure of Potential Conflicts of Interest. Conflicts that the editors consider relevant to the content of the manuscript have been disclosed.
