# Supplementary material for: The Relationship Between Blood Sample Volume and Diagnostic Sensitivity of Blood Culture for Typhoid and Paratyphoid Fever: A Systematic Review and Meta-Analysis
Source: J Infect Dis. 2018 Oct 11;218(Suppl 4):S255–67. doi: 10.1093/infdis/jiy471 (PMC6226661; doi:10.1093/infdis/jiy471)
Supplement: Supplementary Material [file jiy471_suppl_supplementary_material.docx]

**Supplements to: *The relationship between blood sample volume and diagnostic sensitivity of blood culture for typhoid and paratyphoid fever: a systematic review and meta-analysis***

Marina Antillon^1^, Neil J. Saad^1^, Stephen Baker^2,3,4^, Andrew Pollard^5^, Virginia E. Pitzer^1^

*^1^Department of Epidemiology of Microbial Diseases, Yale School of Public Health, New Haven, CT 06520-8034 USA*

*^2^The Hospital for Tropical Diseases, Wellcome Trust Major Overseas Programme, Oxford University, Oxford, United Kingdom*

*^3^Centre for Tropical Medicine, Oxford University, Oxford, United Kingdom*

*^4^Department of Medicine, The University of Cambridge, Cambridge, United Kingdom*

*^5^Oxford Vaccine Group, Department of Paediatrics, University of Oxford and the NIHR Oxford*

*Biomedical Research Centre, Oxford, UK*

Supplement S1: Search strategy 2

Supplement S2: Screening strategy 3

Supplement S3: QUADAS II (risk of bias) tool and data extraction form 4

Supplement S4: Meta-regression analysis. 11

4.1 Specimen volume 11

4.2 Age as a modifier of the relationship between specimen volume and blood culture sensitivity 13

4.3 Interactions between antimicrobial use and duration of symptoms 14

4.4 Software packages 14

Supplement S5: Extended discussion of risk of bias assessment. 14

Supplement S6: Supplementary Tables 17

Supplement S7: Supplementary Figures 30

Supplement S8. Supplement References 36

# Supplement S1: Search strategy

We searched MEDLINE, Embase, Global Health, Web of Science, and PubMed Central for eligible articles. We used free text and controlled vocabulary, except in Web of Science and PubMed Central where this was not available, related to (1) typhoid and paratyphoid and (2) blood or bone marrow. All searches were performed on June 19, 2016 and no date or language restrictions were imposed.

Databases that have controlled vocabulary:

| **MEDLINE** | |
| --- | --- |
| Block 1: Typhoid or paratyphoid in subheadings | Subject headings: exp Salmonella enterica serovar Typhi/ or exp Typhoid Fever/ or exp salmonella enterica serovar paratyphi a/ or exp salmonella enterica serovar paratyphi b/ or exp salmonella enterica serovar paratyphi c/ or exp Paratyphoid Fever/  **AND** |
| Block 2: Blood or bone marrow in subheadings | Subject headings: exp Blood/di, mi or exp Bone Marrow Cells/di, mi or exp Sternum/mi or exp "Bone Marrow"/mi or exp Blood Cells/mi  **AND** |
| Restricted to articles that had the following terms in the title or abstract | ("typhoid" or "typhoidal" or "typhi" or "typhus abdominalis" or "salmonella typhosa" or "paratyphoid" or "paratyphoidal" or "paratyphi" or "salmonella paratyphosa" or "enteric fever" or "enteric fevers").ti,ab |
| **Global Health** | |
| Block 1: Typhoid or paratyphoid in subheadings | Subject headings: exp typhoid/ or exp salmonella typhi/ or exp paratyphoid/ or exp enteric fevers/ or exp salmonella paratyphi/  **AND** |
| Block 2: Blood or bone marrow in subheadings | Subject headings: exp blood sampling/ or exp blood analysis/ or exp blood volume/ or exp blood/ or exp blood specimen collection/ or exp bone marrow/ or exp sternum/  **AND** |
| Restricted to articles that had the following terms in the title or abstract | ("typhoid" or "typhoidal" or "typhi" or "typhus abdominalis" or "salmonella typhosa" or "paratyphoid" or "paratyphoidal" or "paratyphi" or "salmonella paratyphosa" or "enteric fever" or "enteric fevers").ti,ab |
| **Embase** | |
| Block 1: Typhoid or paratyphoid in subheadings | Subject headings: exp Salmonella enterica serovar Typhi/ or exp Typhoid Fever/ or exp Salmonella paratyphi/ or exp salmonella enterica serovar paratyphi a/ or exp salmonella enterica serovar paratyphi b/ or exp *salmonella enterica serovar paratyphi c/ or exp Paratyphoid Fever/  **AND** |
| Block 2: Blood or bone marrow in subheadings | Subject headings: exp bone marrow/ or exp blood/ or exp sternum/ or exp blood sampling/ or exp blood specimen collection/ or exp bone marrow culture/ or blood analysis/di or exp blood culture/ or exp blood volume/ or exp bone marrow examination/  **AND** |
| Restricted to articles that had the following terms in the title or abstract | ("typhoid" or "typhoidal" or "typhi" or "typhus abdominalis" or "salmonella typhosa" or "paratyphoid" or "paratyphoidal" or "paratyphi" or "salmonella paratyphosa" or "enteric fever" or "enteric fevers").ti,ab |

Databases that do not have controlled vocabulary:

| **MEDLINE In-Process and Other Non-Indexed Citations** | |
| --- | --- |
| Block 1: Typhoid or paratyphoid in title. | ("typhoid" OR "typhoidal" OR "typhi" OR "typhus abdominalis" OR "salmonella typhosa" OR "paratyphoid" OR "paratyphoidal" OR "paratyphi" OR "salmonella paratyphosa" OR "enteric fever" OR "enteric fevers").m_title  **AND** |
| Block 2: Blood or bone marrow. Free text in title, abstract, headings, keywords: | ("bone marrow" or "blood" or "sternal marrow" or "bone medulla" or "myeloculture" or "hemoculture").ti,ab,hw,kw |
| **Web of Science core collection** | |
| Block 1: Typhoid or paratyphoid. Free text in title, abstract, author keywords or article keywords: | TI=("typhoid" OR "typhoidal" OR "typhi" OR "typhus abdominalis" OR "salmonella typhosa" OR "paratyphoid" OR "paratyphoidal" OR "paratyphi" OR "salmonella paratyphosa" OR "enteric fever" OR "enteric fevers") OR TS=("typhoid" OR "typhoidal" OR "typhi" OR "typhus abdominalis" OR "salmonella typhosa" OR "paratyphoid" OR "paratyphoidal" OR "paratyphi" OR "salmonella paratyphosa" OR "enteric fever" OR "enteric fevers")  **AND** |
| Block 2: Blood or bone marrow. Free text in title, abstract, author keywords or article keywords: | TI=("bone marrow" or "blood" or "sternal marrow" or "bone medulla" or "myeloculture" or "hemoculture") OR TS=("bone marrow" or "blood" or "sternal marrow" or "bone medulla" or "myeloculture" or "hemoculture") |
| **PubMed Central** | |
| Block 1: Typhoid or paratyphoid in title. | "typhoid"[Title] OR "typhoidal"[Title] OR "typhi"[Title] OR "typhus abdominalis"[Title] OR "salmonella typhosa"[Title] OR "paratyphoid"[Title] OR "paratyphoidal"[Title] OR "paratyphi"[Title] OR "salmonella paratyphosa"[Title] OR "enteric fever"[Title] OR "enteric fevers"[Title]  **AND** |
| Block 2: Blood or bone marrow. Free text in title or abstract | (("bone marrow"[Abstract] OR "sternal marrow"[Abstract] OR "blood"[Abstract])) OR ("bone marrow"[Title] OR "sternal marrow"[Title] OR "blood"[Title]) |

# Supplement S2: Screening strategy

We assessed eligibility by reviewing title and abstract, and subsequently confirmed this by reviewing the full text.

| **Inclusion criteria** |
| --- |
| - The study must be about typhoid and/or paratyphoid fever.  - Blood culture and bone marrow cultures must have been performed on each patient. |
| **Exclusion criteria** |
| - The publication is a review article, a case report, a commentary, or an editorial.  - Blood culture sensitivity is not clearly reported as a proportion of patients who had a bone marrow culture performed.  - Language other than the following: English, Spanish, French, German, Italian. |

#

# Supplement S3: QUADAS II (risk of bias) tool and data extraction form


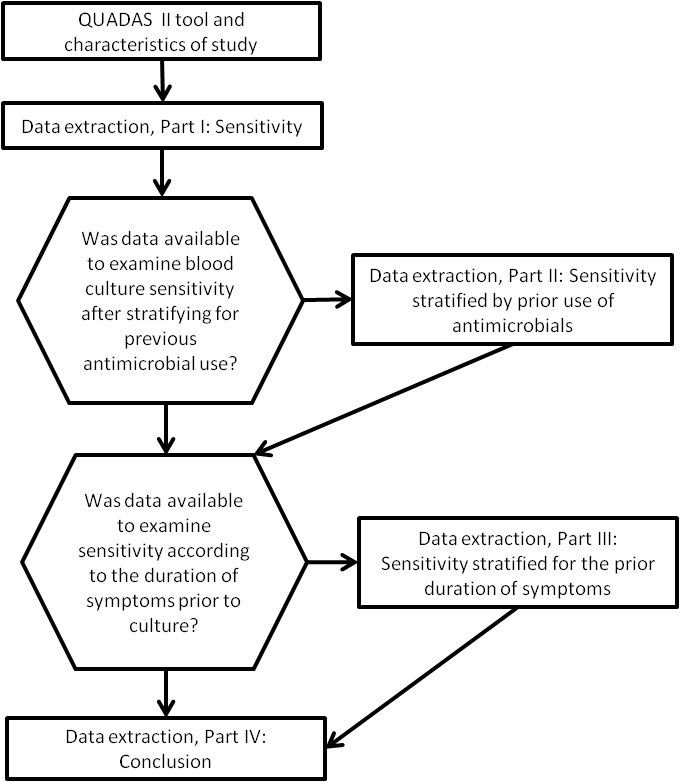


***QUADAS II tool and characteristics of studies:***

Section 1 or 11.

1. Name of the individual performing extraction

2. Last name of the first author of the publication and year of publication

Section 2 of 11. Domain 1: Patient Recruitment.

A. Risk of bias

1. Was the study carried out prospectively (data collected for this purpose) or retrospectively (record review) [Domain 1A]. Choose one of the following

- Prospectively
- Retrospectively
- Other

2. How were the study subjects recruited? [Domain 1A] Choose one of the following:

- At random
- Consecutive
- Unclear

3. Could the selection of patients have introduced bias? [Domain 1A - final question] Choose one of the following:

- Low (only prospective studies)
- High (Retrospective reviews of records)
- Unclear

4. Domain 1A Notes

B. Concerns regarding applicability

1. Where did the study take place ("city, country"; or "unknown")?

2. Was the study carried out in an urban or a rural area center? Choose one of the following:

- Rural
- Urban
- Centers in both settings
- Unclear or not stated

3. What was the median/mean/range of age of the patients included in this study? (Enter "all age groups", specific range, or "unknown"). If you enter the range, specify if the upper or lower bound were designated by design. Please specify whether you are referring to all patients or culture-proven patients only. (open-ended question)

4. Did the authors state the breakdown of patients according to sex or ethnicity? If so, please state it here. Please specify whether you are referring to all patients or culture-proven patients only. (open-ended question)

5. Were patients outpatients, inpatients, a mix? Choose one of the following:

- Inpatient
- Outpatient
- Both
- Unclear or not stated.

6. Is there any indication that the patients received prior treatment (antibiotics, antimicrobials, or other)? If so, explain. (open-ended question)

7. Is there a concern that the included patients differ from patients to whom the diagnostic test will be administered? [Domain 1B - final question] Choose one of the following:

- Low - study performed in a low- to middle-income country, outpatients as well as inpatients
- High - performed on travelers or in a high-income country (Europe, US, Canada)
- High - performed on inpatients
- Unclear

8. Domain 1B Notes (open-ended question)

Section 3 or 11. Domain 2: Index Test (blood culture sensitivity)

A. Risk of Bias

1. How many days was the blood incubated/cultured? (open-ended question)

What broth was used in the blood culture bottle? (open-ended question)

What agar was used to subculture the blood? (open-ended question)

2. Were any of the following strategies to improve detection of *S*. Typhi or *S*. Paratyphi used? Check all that apply:

- Bile salt broth or Oxgall broth used
- Brain-heart infusion (BHI) or tryptic soy broth (TSB) was supplemented with 0.05% sodium polyethanol sulfate (SPS)
- Soybean casein digest broth was used
- Peptone-enriched TSB supplemented with BHI solids
- Inclusion of charcoal of resins in the medium to absorb antimicrobials
- Blood-to-broth dilution ratio: at least 1:10 or more (i.e. 1:12)
- Blood clot, after separation from culture, added to pre-prepared streptokinase broth
- Cells lysed before culture
- Centrifugation of blood followed by direct culture on solid medium
- Blood culture for at least 7 days
- Blood agar (horse or sheep blood) was used to subculture blood
- Nutrient agar and/or MacConkey's agar was used
- Other (please specify)

3. Were the index test results interpreted without knowledge of the results of the reference standard? [Domain 2A] Choose one of the following:

- Yes
- No
- Unclear

4. Could the conduct or interpretation of the index test have introduced bias? [Domain 2A - final question] Choose one of the following:

- Low (blood was incubated & cultured for at least 7 days)
- High (blood was incubated & cultured for less than 7 days)
- Unclear

5. Domain 2A Notes

B. Concerns regarding applicability

1. Is there a concern that the index test, its conduct, or interpretation differ from the review question? [Domain 2B] Marked unclear for all studies - there is no standard way to perform a blood culture for this purpose.

2. Domain 2B Notes

Section 4 of 11. Domain 3: Reference Test(s)

A. Risk of Bias

1. How much bone marrow was collected from each patient? (In milliliters; "not stated" is a valid answer).

2. How many days was the bone marrow cultured? If not stated, write "unspecified".

3. What broth was used in the bone marrow culture bottle?

4. What agar was used to culture the bone marrow?

5. Were the reference test results interpreted without knowledge of the results of the index test? [Domain 3A] Choose one of the following:

- Yes
- No
- Unclear

Could the conduct or interpretation of the reference standard have introduced bias? [Domain 3A - final question]

- Low (>1 mL of bone marrow was cultured)
- High (<1 mL of bone marrow was cultured)
- Unclear

6. Did additional cultures take place? Describe which ones and how the cultures were carried out.

7. Domain 3A Notes

B. Concerns regarding applicability

1. Is there a concern that the reference standard does not adequately identify the target condition? [Domain 3B - final question] Likely to be low for everyone. I had to include the question.

- Low
- High
- Unclear

2. Domain 3B Notes

Section 5 of 11. Domain 4: Flow and timing.

1. Was there any interval between the reference standard and the index test? How long? [Domain 4A] (open-ended question)

2. Did all patients receive the reference standard/index test? If not, did the authors state whether some groups were more likely to receive these than others? [Domain 4A]

- Yes
- No

3. Note: which patients didn't receive both tests

4. Were all patients included in the analysis? If no, who was not included? [Domain 4A]

- Low
- High
- Unclear

5. Note: which patients didn't receive both tests

6. Where were the blood culture and the reference standard(s) cultures carried out? (If stated) (open-ended questions).

7. Could the patient flow have introduced bias? [Domain 4A - final question]

- Low (<1 day interval between blood/bone marrow specimens; all patients were included in the analysis)
- High (1+ day interval between blood/bone marrow specimens or some patients excluded)
- Unclear

8. Domain 4 Notes

***Data extraction***

Section 6 of 11: Part I

Target population: culture confirmed S. Typhi (and S. Paratyphi, if available)

1. Do these results include some patients that have paratyphoid?

- Patients positive for S. Typhi and S. Paratyphi
- Patients positive for S. Typhi alone
- Unclear

2. Enter the blood culture and bone marrow culture results based on small blood sample

|  | **Bone Marrow +** | **Bone Marrow -** | **Blood culture totals** |
| --- | --- | --- | --- |
| **Blood Culture +** |  |  |  |
| **Blood Culture -** |  | Note 1 |  |
| **Bone Marrow totals** |  |  | Grand Total. Note 2. |

Note 1: It is only valid to enter numbers above zero for blood culture -/bone marrow + if additional cultures (i.e. stool, urine, etc).

Note 2: Only cases that are confirmed by at least one culture are counted in this table.

3. How much blood was collected from each patient included in the results in the above table? (In milliliters).

4. What was the midpoint blood volume collected for each patient (i.e. if 3-5 mL of blood were collected, write 4)

5. What was the dilution used for these samples?

6. Enter the blood culture and bone marrow culture results based on larger blood sample

|  | **Bone Marrow +** | **Bone Marrow -** | **Blood culture totals** |
| --- | --- | --- | --- |
| **Blood Culture +** |  |  |  |
| **Blood Culture -** |  | Note 1 |  |
| **Bone Marrow totals** |  |  | Grand Total. Note 2. |

7. How much blood was collected from each patient included in the results in the above table? (In milliliters).

8. What was the midpoint blood volume collected for each patient (i.e. if 3-5 mL of blood were collected, write 4)

9. What was the dilution used for these samples?

10. Section 6 Notes

Section 7 of 11. Part II: sorting question

Sorting question. Was data available to examine blood culture sensitivity after stratifying for previous antimicrobial use?

- Yes. Continue to Section 8.
- No. Continue to Section 9.

Section 8 of 11. Part II: sensitivity stratified for antimicrobial use.

1. Are the results on the impact of antibiotic/antimicrobial use on blood culture sensitivity based on the sample of

- Patients positive for S. Typhi and S. Paratyphi
- Patients positive for S. Typhi alone
- Unclear

2. Enter the blood culture and bone marrow culture results based on small blood sample among individuals who DID NOT use antibiotics or antimicrobials at all.

|  | **Bone Marrow +** | **Bone Marrow -** | **Blood culture totals** |
| --- | --- | --- | --- |
| **Blood Culture +** |  |  |  |
| **Blood Culture -** |  | Note 1 |  |
| **Bone Marrow totals** |  |  | Grand Total. Note 2. |

Note 1: It is only valid to enter numbers above zero for blood culture -/bone marrow + if additional cultures (i.e. stool, urine, etc).

Note 2: Only cases that are confirmed by at least one culture are counted in this table.

3. Enter the blood culture and bone marrow culture results based on small blood sample among individuals who DID NOT use antibiotics or antimicrobials at all.

|  | **Bone Marrow +** | **Bone Marrow -** | **Blood culture totals** |
| --- | --- | --- | --- |
| **Blood Culture +** |  |  |  |
| **Blood Culture -** |  | Note 1 |  |
| **Bone Marrow totals** |  |  | Grand Total. Note 2. |

4. Enter the blood culture and bone marrow culture results based on large blood sample among individuals who DID NOT use antibiotics or antimicrobials at all.

|  | **Bone Marrow +** | **Bone Marrow -** | **Blood culture totals** |
| --- | --- | --- | --- |
| **Blood Culture +** |  |  |  |
| **Blood Culture -** |  | Note 1 |  |
| **Bone Marrow totals** |  |  | Grand Total. Note 2. |

Note 1: It is only valid to enter numbers above zero for blood culture -/bone marrow + if additional cultures (i.e. stool, urine, etc).

Note 2: Only cases that are confirmed by at least one culture are counted in this table.

5. Enter the blood culture and bone marrow culture results based on large blood sample among individuals who DID NOT use antibiotics or antimicrobials at all.

|  | **Bone Marrow +** | **Bone Marrow -** | **Blood culture totals** |
| --- | --- | --- | --- |
| **Blood Culture +** |  |  |  |
| **Blood Culture -** |  | Note 1 |  |
| **Bone Marrow totals** |  |  | Grand Total. Note 2. |

6. Section 8 Notes

Section 9 of 11. Part III: sorting question

Sorting question. Was data available to examine blood culture sensitivity after stratifying for previous antimicrobial use?

- Yes. Continue to Section 10.
- No. Continue to Section 11.

Section 10 of 11. Part III: sensitivity stratified for duration of symptoms before the blood culture took place.

1. Are the results on the impact of antibiotic/antimicrobial use on blood culture sensitivity based on the sample of:

- Patients positive for S. Typhi and S. Paratyphi
- Patients positive for S. Typhi alone
- Unclear

2. Enter the blood culture and bone marrow culture results based on small blood sample among individuals who were experiencing the first week of symptoms.

|  | **Bone Marrow +** | **Bone Marrow -** | **Blood culture totals** |
| --- | --- | --- | --- |
| **Blood Culture +** |  |  |  |
| **Blood Culture -** |  | Note 1 |  |
| **Bone Marrow totals** |  |  | Grand Total. Note 2. |

Note 1: It is only valid to enter numbers above zero for blood culture -/bone marrow + if additional cultures (i.e. stool, urine, etc).

Note 2: Only cases that are confirmed by at least one culture are counted in this table.

3. Enter the blood culture and bone marrow culture results based on large blood sample among individuals who were experiencing the first week of symptoms.

|  | **Bone Marrow +** | **Bone Marrow -** | **Blood culture totals** |
| --- | --- | --- | --- |
| **Blood Culture +** |  |  |  |
| **Blood Culture -** |  | Note 1 |  |
| **Bone Marrow totals** |  |  | Grand Total. Note 2. |

Note 1: It is only valid to enter numbers above zero for blood culture -/bone marrow + if additional cultures (i.e. stool, urine, etc).

Note 2: Only cases that are confirmed by at least one culture are counted in this table.

4. Enter the blood culture and bone marrow culture results based on small blood sample among individuals who were experiencing the second week of symptoms.

|  | **Bone Marrow +** | **Bone Marrow -** | **Blood culture totals** |
| --- | --- | --- | --- |
| **Blood Culture +** |  |  |  |
| **Blood Culture -** |  | Note 1 |  |
| **Bone Marrow totals** |  |  | Grand Total. Note 2. |

Note 1: It is only valid to enter numbers above zero for blood culture -/bone marrow + if additional cultures (i.e. stool, urine, etc).

Note 2: Only cases that are confirmed by at least one culture are counted in this table.

5. Enter the blood culture and bone marrow culture results based on large blood sample among individuals who were experiencing the second week of symptoms.

|  | **Bone Marrow +** | **Bone Marrow -** | **Blood culture totals** |
| --- | --- | --- | --- |
| **Blood Culture +** |  |  |  |
| **Blood Culture -** |  | Note 1 |  |
| **Bone Marrow totals** |  |  | Grand Total. Note 2. |

Note 1: It is only valid to enter numbers above zero for blood culture -/bone marrow + if additional cultures (i.e. stool, urine, etc).

Note 2: Only cases that are confirmed by at least one culture are counted in this table.

6. Enter the blood culture and bone marrow culture results based on small blood sample among individuals who were experiencing the third week of symptoms.

|  | **Bone Marrow +** | **Bone Marrow -** | **Blood culture totals** |
| --- | --- | --- | --- |
| **Blood Culture +** |  |  |  |
| **Blood Culture -** |  | Note 1 |  |
| **Bone Marrow totals** |  |  | Grand Total. Note 2. |

Note 1: It is only valid to enter numbers above zero for blood culture -/bone marrow + if additional cultures (i.e. stool, urine, etc).

Note 2: Only cases that are confirmed by at least one culture are counted in this table.

7. Enter the blood culture and bone marrow culture results based on large blood sample among individuals who were experiencing the third week of symptoms.

|  | **Bone Marrow +** | **Bone Marrow -** | **Blood culture totals** |
| --- | --- | --- | --- |
| **Blood Culture +** |  |  |  |
| **Blood Culture -** |  | Note 1 |  |
| **Bone Marrow totals** |  |  | Grand Total. Note 2. |

Note 1: It is only valid to enter numbers above zero for blood culture -/bone marrow + if additional cultures (i.e. stool, urine, etc).

Note 2: Only cases that are confirmed by at least one culture are counted in this table.

8. Section 10 notes.

Section 11 of 11. Conclusion.

1. Should this study be included in the analyses?

- Yes. Continue to Section 10.
- No. Continue to Section 11.

2. Use the space below to explain why the study does not fit the criteria for inclusion, or to enter any outstanding thoughts about the study. (open-ended questions)

3. Cross-checking references. Any relevant ones we should look at? (open-ended questions)

# Supplement S4: Meta-regression analysis.

## 4.1 Specimen volume

**a. Log-linear regression**

We modeled the probability that a “true positive” patient would test blood-culture positive, $\pi$, given a blood sample of volume *v* (in milliliters). Our analysis aimed to integrate two distinct observational processes present in diagnostic accuracy studies: one set of studies estimated the test sensitivity of patients given a specific volume of blood collected, and a second set of studies tested whether increased sample volumes would yield significantly higher sensitivity by collecting two samples of different volumes from each patient. Standard meta-analytic packages (including those for R, Stata, and Revman) were not capable of integrating probabilities with odds ratios using matched pairs, and thus do not accommodate our analysis and the nature of our data.

Instead, we adopted a hazard approach, in which we modeled the minimum volume of blood necessary to yield a positive sample. Specifically, a cumulative hazard model was selected, as opposed to an ordinary hazards model, because our samples are effectively interval censored: the minimum volume that would yield a positive culture is unobserved. In other words, the probability that a specimen from study *i* of a given volume *v_i,1_* would yield a positive result, $\pi(v_{i,1})$, is equal to the probability that a specimen of that volume or less would be positive:

$\boldsymbol{\pi(}\boldsymbol{v}_{\boldsymbol{i,1}}\boldsymbol{)=}\mathbf{P}\left( \boldsymbol{V}\boldsymbol{\leq}\boldsymbol{v}_{\boldsymbol{i,1}} \right)\mathbf{=}\left[ \mathbf{1}\mathbf{-}\mathbf{exp}\left( \mathbf{-}\boldsymbol{\lambda}_{\boldsymbol{i}\boldsymbol{,1}} \right) \right]$ **(equation 1)**

Our approach thus assumes that drawing a second, larger volume of blood from the same patient whose blood culture was positive given a smaller volume would also yield a positive result (as was observed in the data).

Then we modeled the instances in which smaller samples were culture-negative but larger samples were culture-positive. In this case, the probability that a sample of volume *v* in study *i* between *v_i,1_* (small sample) and *v_i,2_* (larger sample) would yield a positive result, $\pi(v_{i,1}, v_{i,2})$, is a function of the “hazard rates” $\lambda_{i,1}$ and $\lambda_{i,2}$:

$\boldsymbol{\pi(}\boldsymbol{v}_{\boldsymbol{i,1}}\boldsymbol{,}\boldsymbol{v}_{\boldsymbol{i,2}}\boldsymbol{)=}\mathbf{P}\left( \boldsymbol{v}_{\boldsymbol{1}}\mathbf{<}\boldsymbol{V}\boldsymbol{\leq}\boldsymbol{v}_{\boldsymbol{2}} \right)\mathbf{=}\left[ \mathbf{1}\mathbf{-}\mathbf{exp}\left( \mathbf{-}\boldsymbol{\lambda}_{\boldsymbol{i}\boldsymbol{,2}} \right) \right]\boldsymbol{-} \left[ \mathbf{1}\mathbf{-}\mathbf{exp}\left( \mathbf{-}\boldsymbol{\lambda}_{\boldsymbol{i}\boldsymbol{,1}} \right) \right]$ **(equation 2)**

Given these probabilities, the distribution of positive samples in study *i* follow a multinomial distribution:

**Multinomial(**$\boldsymbol{\pi}\left( \boldsymbol{v}_{\boldsymbol{i,1}} \right)\boldsymbol{, \pi(}\boldsymbol{v}_{\boldsymbol{i, 1}}\boldsymbol{,}\boldsymbol{v}_{\boldsymbol{i,2}}\boldsymbol{), 1}\boldsymbol{-}\boldsymbol{\pi}\left( \boldsymbol{v}_{\boldsymbol{i,1}} \right)\boldsymbol{-}\boldsymbol{\pi(}\boldsymbol{v}_{\boldsymbol{i,1}}\boldsymbol{,}\boldsymbol{v}_{\boldsymbol{i,2}}\boldsymbol{)}$**) (equation 3)**

Where $\pi\left( v_{i,1} \right)$ is the probability that each small sample is positive, $\boldsymbol{\pi}\left( \boldsymbol{v}_{\boldsymbol{i, 1}}\boldsymbol{,}\boldsymbol{v}_{\boldsymbol{i,2}} \right)$is the probability that for each patient, the small sample is negative but the large sample is positive, and $1\boldsymbol{-}\boldsymbol{\pi}\left( \boldsymbol{v}_{\boldsymbol{i,1}} \right)\boldsymbol{-}\boldsymbol{\pi(}\boldsymbol{v}_{\boldsymbol{i,1}}\boldsymbol{,}\boldsymbol{v}_{\boldsymbol{i,2}}\boldsymbol{)}$is the probability that both samples from each patient are negative.

For studies in which sensitivity was only estimated using one specimen per person, the likelihood of the observed data becomes a binomial distribution with probability parameter $\pi\left( v_{i,1} \right).$

Three functional forms for the hazard rate were tested:

1. $\boldsymbol{\lambda}_{\boldsymbol{i}}\boldsymbol{=}\boldsymbol{\mu}_{\boldsymbol{i}}$
2. $\boldsymbol{\lambda}_{\boldsymbol{i}}\boldsymbol{=}\boldsymbol{\beta}_{\boldsymbol{i}}\boldsymbol{v}_{\boldsymbol{i}}$
3. $\boldsymbol{\lambda}_{\boldsymbol{i}}\boldsymbol{=}\boldsymbol{\mu}_{\boldsymbol{i}}\boldsymbol{+}\boldsymbol{\beta}\boldsymbol{v}_{\boldsymbol{i}}$

These are described in detail below.

Functional form (A) assumes that there is no effect of blood volume on culture sensitivity and all variance between studies is modeled as a random effect. For this functional form, we note that $\lambda_{i,2}=2\mu_{i}$ regardless of the difference in volume between the first and second sample because the probability that neither sample is positive is $\exp\left( -\mu_{i} \right) \times\exp\left( -\mu_{i} \right)= \exp\left( -{2\mu}_{i} \right)$ and the probability that at least one of two samples is positive is $1-\exp(-2\mu_{i})$. It follows that the probability that the first sample is negative and the second sample is positive is equal to the difference in probability that both samples are negative and the probability that first sample is negative:

$\boldsymbol{\pi(}\boldsymbol{v}_{\boldsymbol{i,1}}\boldsymbol{,}\boldsymbol{v}_{\boldsymbol{i,2}}\boldsymbol{)=}\mathbf{P}\left( \boldsymbol{v}_{\boldsymbol{1}}\mathbf{<}\boldsymbol{V}\boldsymbol{\leq}\boldsymbol{v}_{\boldsymbol{2}} \right)\mathbf{=exp}\left( \mathbf{-}\boldsymbol{\mu}_{\boldsymbol{i}} \right) \mathbf{-}\mathbf{exp}\left( \mathbf{-}\mathbf{2}\boldsymbol{\mu}_{\boldsymbol{i}} \right)$**,**

which is equivalent to $\left[ 1-\exp\left( -\lambda_{i,2} \right) \right]- \left[ 1-\exp\left( -\lambda_{i,1} \right) \right]$ and analogous to equation 2, above.

Functional form (B) assumes that there is a log-linear effect, ***β***, of blood volume on sensitivity, and that the blood culture sensitivity for a hypothetical 0 mL sample is 0; we again modeled the term ***β*** as a random effect to account for between-study heterogeneity. Functional form (C) also assumes that there is a log-linear relationship between blood volume and sensitivity, but we estimate an additional term, *µ*, for the theoretical sensitivity of a 0 mL sample, and a slope, ***β***, for the incremental gain in sensitivity that would result from collecting an additional mL of blood. To account for heterogeneity across studies, we modeled the term *µ* as a random effect using the study as a group variable. All random effects were assumed to derive from a log-normal distribution with shared hyper-parameters (estimated).

We compared the three models using the Widely Applicable Information Criterion (WAIC). We considered a model to provide a better fit if it yielded a lower WAIC.

The relationship between sensitivity and blood specimen volume was attenuated in the best-fit model when we considered bone marrow culture-positive patients (rather than any culture-positive patients) as the reference group. To better understand why this occurred, we re-ran the analysis among the subset of studies (n=22) that reported blood culture positivity specifically among patients who were positive by bone marrow culture (Table S2 and Figure S7). The relationship with sample volume was again attenuated in Model C in this analysis, although the baseline sensitivity (intercept) was higher. However, we found no systematic difference between estimates of blood culture sensitivity based on the reference standard (Figure S8). Thus, we believe the attenuated relationship with blood volume in this analysis can primarily be attributed to the exclusion of three studies (Hoffman 1984, Hoffman 1986, Vallenas 1985), and in particular one study (Hoffman 1986) that measured sensitivity in samples of two different volumes, which is especially important when including a random effect on the slope parameter.

**b. Linear regression**

We also examined the sensitivity of our results to the model structure by comparing our results from the log-linear functional form described above (4.2.a) to those from a simple linear regression model, in which the probability that a sample of volume *v_i,1_* would yield a positive result, $\pi(v_{i,1})$, is given by:

$$\boldsymbol{\pi(}\boldsymbol{v}_{\boldsymbol{i,1}}\boldsymbol{)=}\left\{ \begin{aligned} \boldsymbol{\mu}_{\boldsymbol{i}}\boldsymbol{+}\boldsymbol{\beta}\boldsymbol{v}_{\boldsymbol{i}} \text{if} \boldsymbol{\mu}_{\boldsymbol{i}}\boldsymbol{+}\boldsymbol{\beta}\boldsymbol{v}_{\boldsymbol{i}}\boldsymbol{<1} \\ \boldsymbol{1}\text{otherwise} \end{aligned} \right.$$

However, this functional form does not take into account the fact that some studies took two samples from each patient, nor the fact that the larger specimens were always positive among patients who had positive blood culture results from smaller specimens. The results of this model are shown below (Figure S3 and Table S6). Note that the relationship with blood volume did not vary based on the reference group (all culture-positive patients versus bone marrow culture-positive patients only) in this analysis.

## 4.2 Age as a modifier of the relationship between specimen volume and blood culture sensitivity

In order to assess the impact of age on the relationship between specimen volume and diagnostic sensitivity, we added age group to the model (C) chosen as the best-fit model in the above analysis:

**D.** $\boldsymbol{\lambda}_{\boldsymbol{i}}\boldsymbol{=}\boldsymbol{\mu}_{\boldsymbol{i}}\boldsymbol{+}\boldsymbol{\beta}\boldsymbol{v}_{\boldsymbol{i}}\boldsymbol{+}\boldsymbol{\gamma}_{\boldsymbol{a}}$

**E.** $\boldsymbol{\lambda}_{\boldsymbol{i}}\boldsymbol{=}\boldsymbol{\mu}_{\boldsymbol{i}}\boldsymbol{+}\boldsymbol{\beta}\boldsymbol{v}_{\boldsymbol{i}}\boldsymbol{+}\boldsymbol{\gamma}_{\boldsymbol{a}}\boldsymbol{+}\boldsymbol{\psi}_{\boldsymbol{a}}\boldsymbol{v}_{\boldsymbol{i}}$

where *γ_a_* is the independent effect of age group *a* on sensitivity, and $\psi_{a}$ is the interaction (effect modification) of age on the relationship between volume and sensitivity.

We tested two parameterizations. Model (D) evaluated the effect of age as an independent predictor of sensitivity and potential confounder of the relationship between specimen volume and blood culture sensitivity. Model (E) evaluated the effect of age as an independent predictor and as a modifier of the relationship between specimen volume and sensitivity; effectively, this models a different relationship between specimen volume and sensitivity for each age group. However, because of a lack of data, model (E) did not converge.

Studies were categorized into the following age groups: children (the referent age group), older children and adults, adults only, all ages, and a category of "unclear" to designate those studies that did not report the age range of study participants. Although the age-related parameters corresponding to the categories of "all ages" and "unclear" are not interpretable, we retained the corresponding studies to facilitate estimation of parameters related to the specimen volume.

We compared models using the Widely Applicable Information Criterion (WAIC) and we considered a model to provide a better fit if it yielded a lower WAIC.

## 4.3 Interactions between antimicrobial use and duration of symptoms

We explored the potential interaction between symptom duration and antimicrobial use by employing two approaches. First, we examined the effects of symptom duration in the pre-antibiotic era and the antibiotic era via subgroup analysis. Second, we leveraged the patient-level data provided in one study (Seshadri 1977) [1] that reported person-level data on the duration of illness and antimicrobial use, making it possible to test the joint effect of these two factors to sensitivity. We built a logistic model and used the likelihood ratio test to assess the statistical significance of including antimicrobial use and duration of symptoms as independent and joint predictors of diagnostic sensitivity.

## 4.4 Software packages

All analyses were performed using R 3.2.2 using the package meta [2] and metafor [3]; generalized linear models were implemented using the package lme4 [4]; and likelihood ratio tests were performed via the package lmtest [5]. Meta-regression analyses were performed using the JAGS (Just Another Gibbs Sampler) software, version 3.4.0, in conjunction with the R interface for JAGS, rjags [6,7]. Graphics were produced using ggplot [8].

# Supplement S5: Extended discussion of risk of bias assessment.

We modified the QUADAS II tool to fit our research question and assessed the risk of bias in patient recruitment, index test (blood culture), reference test (bone marrow culture), or the flow and timing of the two cultures.

*Representative patient spectrum.* The QUADAS II tool recommends that the patient recruitment strategy be taken into account in the risk of bias, but none of our studies reported whether patients were recruited at random or consecutively (as a “convenience” sample). Therefore, all our studies were considered unclear in this respect.

In assigning a risk of bias score, we determined that prospective studies were at a low risk of bias and that retrospective studies (based on a record review) were at a high risk of bias because of the potential to select a non-representative sample of the patients. Based on this criterion, seven studies were retrospective reviews and were accorded a high risk of bias, one study did not state whether it was retrospective or prospective, and all other studies were considered to be at a low risk for bias (Table S5).

We assigned an applicability score (of high or low) based on whether or not the study was carried out in a present-day low- or middle-income country (LMIC) and whether or not both outpatients and inpatients were recruited to the study. Two studies were considered at low risk of bias in terms of applicability, 13 studies were unclear (because it was unclear if the study included both inpatient and outpatient populations), and 25 studies were considered at high risk of bias for the following reasons; 19/25 studies were carried out in inpatient populations, 4/25 studies were performed in North America or Europe, and 2/25 studies were performed among inpatients in a non-LMIC setting (Table S5).

*Implementation of index tests.* The implementation of blood culture techniques was often not clear, and therefore most risk of bias and applicability scores were marked unclear. For instance, with two exceptions, it was not known whether the index tests were carried out without knowledge of the reference test (bone-marrow culture). We chose to assign a risk of bias score according to the length of incubation, which was based on the literature. If the blood sample was incubated and cultured for 7 days or more, we considered that it was a “low” risk of bias [9,10]. Twenty-seven studies were unclear on the culture procedure, eight studies had low risk of bias, and five studies had a high risk of bias (Table S5). No study stated how cultures were screened for contamination. Some studies employed additional strategies to increase the sensitivity of the blood sample; these details are in Table S1. We considered all studies to be “unclear” in terms of applicability because there is no standardized protocol exists for the purpose of typhoid diagnosis despite the heterogeneity attributable to laboratory techniques documented in the literature [11].

*Implementation of reference test.* To date, cultures consisting of bone marrow extracted from the sternum or the iliac crest are considered the gold standard diagnostic for typhoid fever, but there is officially no standard protocol for bone-marrow cultures. Therefore, we scored the risk of bias based solely on whether or not the sample of bone marrow was smaller than 1 mL (high risk) or greater than or equal to 1 mL (low risk). We determined that there were low applicability concerns with respect to the reference test (bone marrow culture) because it has high sensitivity [11,12].

All but three of the studies stated whether the results of the bone marrow (the reference standard) were recorded without knowledge of the blood culture results (the index test) or vice versa, which could hypothetically influence study-specific estimates of sensitivity, but since these are culture studies, misinterpretation is unlikely. Moreover, none of the studies in the analysis stated how samples were screened for contamination.

*Patient recruitment and flow.* We assessed the risk of bias in patient recruitment and flow based on whether the blood and bone marrow samples were taken simultaneously or within the same day, and whether all patients were included in the analysis. Twenty-four studies did not state whether the blood and bone marrow samples were taken simultaneously or within the same day. Two studies stated that both samples were taken before treatment was initiated, even though it was not clear how close in time the samples were taken. Of the remaining 14 studies, all but two studies collected the samples within one day.

Out of 40 studies, 28 studies collected samples from all patients and one study was unclear, and 27 studies included all sample donors in the analysis, eight studies did not include all donors in the analysis, and five other studies were unclear in this regard. Table S5 provide details on the reasons for which some patients were excluded from the analysis (see notes in table), but the main reason patients were excluded from the reported results was that another diagnostic test of interest in the study (e.g. rose-spot culture, duodenal string device) was not performed.

# Supplement S6: Supplementary Tables

**Table S1. Culture description.**

| **Study (language other than English)** | **Urban or rural location** | **Sex distribution of the sample** | **Antimicrobial use** | **Strategies employed to improve detection of S. typhi used?** | **Volume of the bone marrow specimen** | **How long was the bone marrow culture incubated?** | **What broth was used to culture the bone marrow specimen?** | **What agar was used to subculture the bone marrow specimen?** |
| --- | --- | --- | --- | --- | --- | --- | --- | --- |
| Akoh 1991 [13] | Urban | Not reported. | Among 11 BM+/BC- cases, 6 patients had previous exposure to antibiotics. Among 9 BM-/BC- cases, 6 patients had previous exposure to antibiotics. Distribution not stated among BC+. |  | 1-2mL | Not reported. | Thioglycolate. | Not reported. |
| Avendano 1986[14] | Urban | Not reported. | Not reported. | b, f | Not reported. | Not reported. | Brain heart infusion with sodium polyanetholsulfonate. | *Salmonella*-*Shigella*, bismuth sulfite, Kliglers triple sugar iron. |
| Baqi Durrani 1996 [15] | Urban | Among 204 patients, 130 were males and 74 were females. However, only 95 had blood and bone marrow cultures performed, and we don't know the ratio of men to women. | Data was extracted for analysis. Among 95 culture-confirmed cases, 70 had previous exposure to antibiotics. | j, k, l | 1 mL | 7 days | Brain heart infusion. | Sheep's blood, MacConkey's, *Salmonella*-*Shigella* agar plates. |
| Barbagallo 1938 (Italian) [16] | Urban | Not reported. | Antibiotics were not in use at this time, and use of other antimicrobials was not stated. | l | Not reported. | Not reported. | Oxoid (trypticase-soy). | MacConkey's, Oxoid (trypticase-soy broth), and triple sugar iron agar. |
| Bassily 1980*[17] | Rural | All male. | Not reported. | a | 4-5 mL | Not reported. | Bile | Not reported. |
| Benavente 1981[18] | Urban | Not reported. | Among 20 culture-confirmed cases, 12 had previous exposure to antibiotics. | a | Not reported. | Not reported. | Oxgall (oxbile). | Not reported. |
| Benavente 1984[19] | Urban | Both sexes but no exact data reported. | Not reported. |  | 0.5-1 mL | >48 hrs | Oxgall (oxbile). | *Salmonella*-*Shigella* |
| Bhutta 1991*[20] | Urban | Not reported. | Not reported. |  | Not reported. | Not reported. | Not reported. | Not reported. |
| Chaicumpa 1992 [21] | Urban | Not reported. | Not reported. | j,l | 0.5-0.8 mL | 7 Days | Oxgall (oxbile). | MacConkey's, *Salmonella*-*Shigella,* and BBL (trypticase-soy). |
| Chang 1982 (Spanish)* [22] | Urban | Not reported. | Data not reported for the subset of patients that had both cultures performed. |  | Not reported. | Not reported. | Not reported. | Not reported. |
| **Study (language other than English)** | **Urban or rural location** | **Sex distribution of the sample** | **Antimicrobial use** | **Strategies employed to improve detection of S. typhi used?** | **Volume of the bone marrow specimen** | **How long was the bone marrow culture incubated?** | **What broth was used to culture the bone marrow specimen?** | **What agar was used to subculture the bone marrow specimen?** |
| Chiragh 2005* [23] | Rural | Among 50 patients 37 were male and 13 were female. Not all of these patients were cultured. | People with previous exposure to antibiotics were excluded. |  | Not reported. | Not reported. | Not reported. | Not reported. |
| Dance 1991 [24] | Urban | Not reported. | Among 3 patients who were BM+/BC-, 2 had previous exposure to antibiotics, but the distribution among BM+/BC+ cases was not reported. | b, f | 0.5-1 mL | Not reported. | Brain heart infusion containing liquoid. | Not reported. |
| Debre 1935 (French)* [25] | Urban | Among 7 culture-confirmed cases, 4 were male and 3 were female. | Not reported. | a | Not reported. |  | Oxgall (oxbile). | Not reported. |
| Del Negro 1960 (Portuguese)* [26] | Unclear | Among 60 suspected cases of typhoid fever, 35 were male and 25 were female. | Not reported. |  | Not reported. | Not reported. | Not reported. | Not reported. |
| Farooqui 1991 [27] | Urban | Not reported. | Not reported. | j, f, k, l | 0.5-1 mL | 7 days | Brain heart infusion and thioglycolate. | MacConkey'sand blood agar |
| Gasem 1995 [28] | Urban | Among 145 suspected typhoid cases, 75 were male and 70 were female. Only 86 cases were culture-confirmed. | Among 145 suspected typhoid cases, 117 cases had previous exposure to antibiotics. | a, f, j | 1 mL | 7 days | Oxgall (oxbile). | *Salmonella*-*Shigella*; suspected cultures were subcultured on Triple Sugar Iron agar. |
| Gasem 2003 [29] | Urban | Among 100 patients considered for the study, 53 male and 47 female | Patients were excluded for previous exposure of chloramphenicol, ciprofloxacin, or quinolones. |  | 5 mL | Not reported. | Not reported. | Cultured with BACTEC 9120 |
| Gilman 1975 [30] | Urban | Not stated | Not reported. |  | Not reported. | Not reported. | Peptone and Ruiz-Castaneda broth | Not reported. |
| Guerra-Caceres 1979 [31] | Urban | Among 60 suspected typhoid cases, 36 were male and 24 female. | Data was extracted for analysis. Among 57 culture-confirmed cases, 34 had previous exposure to antibiotics. All 3 BM-/BC- patients had previous exposure to antibiotics. | j | 0.5 mL | 10 days | Oxoid (trypticase-soy) for half the specimens, and Ruiz-Castaneda for the other half. | Not reported. |
| Hirsowitz 1951* [32] | Urban | Not reported. | Not reported. | l | Not reported. | Not reported. | Oxgall (oxbile). | Not reported. |
| Hoffman 1984 [10] | Urban | Among 154 suspected typhoid cases, 87 were male and 67 were female. | Patients may have received antibiotics for up to 2.5 days prior to culture collection, but no further details were reported. | a, j, l | 0.5-0.8 mL | 7 Days | Oxgall (oxbile). | MacConkey, Salmonella Shigella and BBL (trypticase-soy). |
| Hoffman 1986 [33] | Urban | Among culture-proven cases, 29 were male and 32 were female. | Data was extracted for analysis. | a, f, j | 0.5-0.8 mL | 21 days | Oxgall (oxbile). | Not reported. |
| **Study (language other than English)** | **Urban or rural location** | **Sex distribution of the sample** | **Antimicrobial use** | **Strategies employed to improve detection of S. typhi used?** | **Volume of the bone marrow specimen** | **How long was the bone marrow culture incubated?** | **What broth was used to culture the bone marrow specimen?** | **What agar was used to subculture the bone marrow specimen?** |
| James 1997* [34] | Urban | Among culture-proven cases, 25 were male and 11 were female. | Patients on chloramphenicol or other bone marrow depressants were excluded. No further details were reported. |  | Not reported. | Not reported. | Not reported. | Not reported. |
| Ling 1940 [35] | Urban | Not reported. | Antibiotics were not in use at this time, and use of other antimicrobials was not stated. |  | 0.5 mL | Not reported. | Meat liver. | Not reported. |
| Ling 1948 [36] | Urban | Not reported. | Antibiotics were not in wide use at this time, and use of other antimicrobials was not stated. | j | 0.66 mL | 7 days | Oxgall (oxbile) and peptone. | Endo's medium. |
| Mehta 1984 [37] | Urban | Among 50 patients, 35 were male and 15 were female. | Among 50 patients of suspected typhoid, 13 patients had previous exposure to antibiotics. | a, f, l | 0.5 mL | 2 days | Oxgall (oxbile). | MacConkey's. |
| Ott 1938 (German)* [38] | Urban | Not reported. | Antibiotics were not in use at this time, and use of other antimicrobials was not stated. |  | Not reported. | Not reported. | Oxgall (oxbile). | Not reported. |
| Rajagopal 1986 [39] | Urban | Not reported. | Data was extracted for analysis. Exposure to antibiotics was defined as anyone who had "swallowed 2-4 capsules for a few days." | a | 0.5 mL | Not reported. | Oxgall (oxbile). | Not reported. |
| Rubin 1989 [40] | Urban | Not reported. | Not reported. | j, l | Not reported. | 7 Days | Oxgall (oxbile). | MacConkey's, *Salmonella-Shigella* and BBL (trypticase-soy). |
| Sacks 1941* [41] | Urban | Among culture-proven cases, 1was male and 2 were female. | Antibiotics were not in use at this time, and use of other antimicrobials was not stated. |  | 4 mL | Not reported. | Not reported. | Not reported. |
| Schlack 1966 (Spanish) [42] | Urban | Not reported. | Among 103 BM+ cases 45 had previous exposure to antibiotics treatment and among 53 BC+ cases, 23 had prior antibiotic treatment. There were 107 total culture-confirmed cases. |  | 2 mL | 1 day | Meat liver. | Not reported. |
| Seidenstucker 1949 (German) [43] | Urban | Not reported. | Not reported. |  | Not reported. | Not reported. | Not reported. | Not reported. |
| Sekarwana 1989 [44] | Urban | Among 28 culture-proven cases, 15 were male and 13 were female. | Data was extracted for analysis. Among 28 culture-confirmed cases, 12 had received previous treatment. | j | 0.5-1 mL | 7 days | Oxgall (oxbile). | *Salmonella-Shigella.* |
| Seshadri 1977 [45] | Urban | Not reported. | Data was extracted for analysis. Among culture-confirmed cases, 14 had previous exposure to antibiotics. | a, l | 0.5 mL | 48 hours of incubation, days of culturing were not stated. | Oxgall (oxbile). | MacConkey's |
| **Study (language other than English)** | **Urban or rural location** | **Sex distribution of the sample** | **Antimicrobial use** | **Strategies employed to improve detection of S. typhi used?** | **Volume of the bone marrow specimen** | **How long was the bone marrow culture incubated?** | **What broth was used to culture the bone marrow specimen?** | **What agar was used to subculture the bone marrow specimen?** |
| Shin 1994* [46] | Urban | Among culture-proven cases, 7 were men and 8 were female. | Among 15 culture-confirmed cases, 11 cases had prior exposure to antibiotics. |  | Not reported. | Not reported. | Not reported. | Not reported. |
| Storti 1937 (French)* [47] | Urban | Not reported. | Antibiotics were not in use at this time, and use of other antimicrobials was not stated. |  | Not reported. | Not reported. | Not reported. | Not reported. |
| Terminel 1973 (Spanish)* [48] | Urban | Not reported. | Not reported. |  | Not reported. | Not reported. | Not reported. | Not reported. |
| Vallenas 1985 [49] | Urban | Among culture-proven cases, 22 were female and 21 were male. | Data was extracted for analysis. | a | 0.5 mL | Not reported. | Oxgall (oxbile). | Not reported. |
| Wain 2008 [50] | Centers in both settings | Not reported. | Not reported. | a, b, i, j, k | At least 1 mL | Not reported. | Not reported. | Not reported. |
| West 1989* [51] | Urban | Among culture-proven patients, 54 were male and 38 female. | Not reported. | j | Not reported. | 3 days and then weekly for 3 weeks | Oxgall (oxbile). | Not reported. |

*Indicates that the study will only be included in the summary of the systematic review, but not in the analysis examining blood sample volume and sensitivity.

These were the techniques to improve blood culture sensitivity that we looked for in each study [52–55]: a) Bile salt broth or Oxgall broth used; b) Brain-heart infusion (BHI) or tryptic soy broth (TSB) was supplemented with 0.05% sodium polyethanol sulfate (SPS); c) Soybean casein digest broth was used; d Peptone-enriched TSB supplemented with BHI solids; e) Inclusion of charcoal of resins in the medium to absorb antimicrobials; f) Blood-to-broth dilution ratio: at least 1:10 or more (i.e. 1:12); g) Blood clot, after separation from culture, added to pre-prepared streptokinase broth; h) Cells lysed before culture; i) Centrifugation of blood followed by direct culture on solid medium; j) Blood culture for at least 7 days; k) Blood agar (horse or sheep blood) was used to subculture blood; l) Nutrient agar and/or MacConkey's agar was used.

**Table S2. Model parameter estimates and Widely Applicable Information Criterion (WAIC) scores.** We show the model parameter estimates as well as the goodness-of-fit measures (WAIC) for the meta-regression models of the relationship between specimen volume and blood culture sensitivity among patients with at least one culture-positive specimen and among bone-marrow culture positive patients only. For parameters that account for random effects, we also report the mean and variance hyper-parameters.

|  | **Among any culture-positive samples**  **(25 studies)** | **Bone marrow positive**  **(22 studies)** | **Among any culture-positive samples in studies that also had data for the analysis in column 2 (22 studies)** |
| --- | --- | --- | --- |
| ***Model A*** |  |  |  |
| **Intercept parameter (*μ*)** | 0.77 (0.66, 0.91) ^a^ | 0.79 (0.64, 0.96) ^b^ | 0.80 (0.66, 0.95) ^g^ |
| **WAIC** | 193.1 | 156.7 | 168.7 |
| ***Model B*** |  |  |  |
| **Slope parameter (*β*)** | 0.19 (0.16, 0.22) ^c^ | 0.19 (0.15, 0.22) ^d^ | 0.16 (0.19, 0.22)^h^ |
| **WAIC** | 239.83 | 188.0 | 210.9 |
| ***Model C*** |  |  |  |
| **Intercept parameter (*μ*)** | 0.63 (0.47, 0.78) ^e^ | 0.78 (0.62, 0.95) ^f^ | 0.63 (0.78, 0.95)^i^ |
| **Slope parameter (*β*)** | 0.04 (0.02, 0.07)* | 0.005 (0.0002, 0.03)* | 0.005 (0.0002, 0.02)* |
| **WAIC** | 174.2 | 128.0 | 130.0 |

*In Model C, which includes both an intercept and a slope, we only account for random effects in the intercept

^a^ Parameters for the lognormal distribution: Mean: -0.26 (-0.42, -0.10), SD: 8.62 (3.87, 19.47)

^b^ Parameters for the lognormal distribution: Mean: -0.24 (-0.45, -0.05), SD: 6.44 (2.76, 14.39)

^c^ Parameters for the lognormal distribution: Mean: -1.66 (-1.81, -1.51), SD: 11.30 (4.84, 27.33)

^d^ Parameters for the lognormal distribution: Mean: -1.69 (-1.87, -1.51), SD: 9.04 (3.73, 22.23)

^e^ Parameters for the lognormal distribution: Mean: -0.47 (-0.76, -0.25), SD: 6.85 (2.76, 17.24)

^f^ Parameters for the lognormal distribution: Mean: -0.25 (-0.49, -0.05), SD: 6.64 (2.77, 15.54)

^g^ Parameters for the lognormal distribution: Mean: -0.23 (-0.41, -0.05), SD: 8.02 (3.39, 18.81)

^h^ Parameters for the lognormal distribution: Mean: -1.67 (-1.83, -1.50), SD: 11.25 (4.42, 31.08)

^i^ Parameters for the lognormal distribution: Mean: -0.24 (-0.46, -0.05), SD: 7.52 (3.13, 17.87)

**Table S3. Age as a potential confounder of the relationship between blood culture sensitivity and specimen volume.**

|  | **Among any culture-positive samples** |
| --- | --- |
| ***Model D*** |  |
| **Intercept parameter (*μ*)** | 0.59 (0.32, 0.88) |
| **Slope parameter (*β*)** | 0.04 (0.03, 0.07) |
| **Age group parameters (*γ*)** |  |
| **Children** | Referent |
| **Older Children and Adults** | -0.17 (-0.51, 0.14) |
| **Adults** | 0.29 (-0.21, 0.72) |
| **All ages** | -0.02 (-0.41, 0.31) |
| **Unclear** | 0.03 (-0.26, 0.32) |
| **WAIC** | 174.7 |

Parameters for the lognormal distribution: Mean: -0.52 (-1.14, -0.13), SD: 7.77 (1.82, 25.52)

**Table S4. Impact of duration of symptoms and use of chloramphenicol prior to culture on blood culture sensitivity using person-level data featured in Seshadri, et al 1977.** We tested the effect of prior duration of symptoms and previous chloramphenicol treatment on blood culture sensitivity using the likelihood ratio test. Model comparisons 1 and 2 test the impact of the prior duration of symptoms on sensitivity by parameterizing the variable for duration of symptoms as a linear relationship and a quadratic relationship. Model comparison 3 tests the impact of previous chloramphenicol treatment on sensitivity. Model comparison 4 tests the effect of chloramphenicol treatment as a mediator of the relationship between duration and sensitivity. Model comparison 5 tests the relationship of duration as a confounder of the relationship between chloramphenicol treatment and sensitivity. Model comparison 6 tests the modifying effect of (or the interaction between) previous chloramphenicol treatment on the relationship between previous duration of symptoms and sensitivity.

| Model comparison number | Model comparison | p-value (likelihood ratio test) |
| --- | --- | --- |
| 1 | logit(positive culture) ~ Intercept *+ X_D_* vs  logit(positive culture) ~ Intercept | 0.70 |
| 2 | logit(positive culture) ~ Intercept *+ X_D_* + *X_D_^2^* vs  logit(positive culture) ~ Intercept *+ X_D_* | 0.46 |
| 3 | logit(positive culture) ~ Intercept *+ X_A_* vs  logit(positive culture) ~ Intercept | 0.40 |
| 4 | logit(positive culture) ~ Intercept *+* *X_D_* + *X_A_* vs logit(positive culture) ~ Intercept *+ X_D_* | 0.39 |
| 5 | logit(positive culture) ~ Intercept *+ X_D_* + *X_A_* vs logit(positive culture) ~ Intercept *+ X_A_* | 0.68 |
| 6 | logit(positive culture) ~ Intercept *+ X_D_* + *X_A_* + *X_D_X_A_* vs logit(positive culture) ~ Intercept *+ X_D_* + *X_A_* | 0.06 |
| 7 | logit(positive culture) ~ Intercept *+ X_D_* + *X_A_* + *X_D_X_A_* vs logit(positive culture) ~ Intercept | 0.21 |

*X_A_* = Indicator variable for antimicrobial use prior to blood culture.

*X_D_* = Variable for duration of symptoms prior to blood culture.

**Table S5. Risk of bias assessment.**

|  |  | **Patient selection** | | | **Index test** | | | **Reference standard** | | | **Flow and timing** | | | |
| --- | --- | --- | --- | --- | --- | --- | --- | --- | --- | --- | --- | --- | --- | --- |
| **Study** | **Study design** | **Patient recruitment** | **Risk of bias** | **Applicability concerns (reasons in parenthesis, see notes for abbreviations)** | **Blinding** | **Risk of bias** | **Applicability concerns** | **Blinding** | **Risk of bias** | **Applicability concerns** | **Interval between tests** | **All patients included in testing?** | **All patients included in analysis?** | **Flow and timing, risk of bias** |
| Akoh 1991 [13] | Prospective | Unclear | Low | High (IP) | Unclear | Unclear | Unclear | Unclear | Low | Low | Not reported | No | Yes | Unclear |
| Avendano 1986 [14] | Prospective | Unclear | Low | High (IP) | Unclear | Unclear | Unclear | Unclear | Unclear | Low | Not reported | Yes | Yes | Unclear |
| Baqi Durrani 1996 [15] | Prospective | Unclear | Low | Low | Unclear | Low | Unclear | Unclear | Low | Low | Taken simultaneously. | No (because of lack of resources) | Yes | Low |
| Barbagallo 1938 (Italian) [16] | Unclear | Unclear | Unclear | High (IP, HI) | Unclear | Unclear | Unclear | Unclear | Low | Low | Taken simultaneously. | Yes | Yes | Low |
| Bassily 1980* [17] | Prospective | Unclear | Low | High (IP) | Unclear | Unclear | Unclear | Unclear | Unclear | Low | Same day. | Yes | Yes | Low |
| Benavente 1981 [18] | Prospective | Unclear | Low | Low | Unclear | Unclear | Unclear | Unclear | Unclear | Low | Same day. | Yes | Yes | Low |
| Benavente 1984 [19] | Prospective | Unclear | Low | Unclear | Unclear | Unclear | Unclear | Unclear | High | Low | Not reported | Yes | Yes | Unclear |
| Bhutta 1991* [20] | Retrospective | Unclear | High | Unclear | Unclear | Unclear | Unclear | Unclear | Unclear | Low | Taken simultaneously. | No | Yes | Low |
| Chaicumpa 1992 [21] | Prospective | Unclear | Low | High (IP) | Unclear | Unclear | Unclear | Unclear | Unclear | Low | Not reported | Yes | Yes | Unclear |
| Chang 1982 (Spanish)* [22] | Retrospective | Unclear | High | Unclear | Unclear | Unclear | Unclear | Unclear | Unclear | Unclear | Not reported | No | Yes | Unclear |
| Chiragh 2005* [23] | Prospective | Unclear | Low | Unclear | Unclear | Unclear | Unclear | Unclear | Unclear | Low | Taken simultaneously. | Yes | Yes | Low |
| Dance 1991 [24] | Prospective | Unclear | Low | Unclear | Unclear | Unclear | Unclear | Unclear | High | Low | Not reported | Yes | Unclear | High |
| Debre 1935 (French)* [25] | Retrospective | Unclear | Unclear | High (HI) | Yes | Unclear | Unclear | No | High | Low | Between 0 and 4 days apart. | Unclear | Unclear | High |
| Del Negro 1960 (Portuguese)* [26] | Retrospective | Unclear | High | High (IP) | Unclear | Unclear | Unclear | Unclear | Unclear | Low | Not reported | No | Yes | Unclear |
| Farooqui 1991 [27] | Retrospective | Unclear | High | Unclear | Unclear | Low | Unclear | Unclear | High | Low | Not reported | No | Unclear | Unclear |
| Gasem 1995 [28] | Prospective | Unclear | Low | High (IP) | Unclear | Low | Unclear | Unclear | Low | Low | Not reported | Yes | Yes | High |
| Gasem 2003 [29] | Prospective | Unclear | Unclear | High (IP) | Unclear | Unclear | Unclear | Unclear | Low | Low | Not reported | Yes | No | Unclear |
| Gilman 1975 [30] | Prospective | Unclear | Low | Unclear | Unclear | Unclear | Unclear | Unclear | Unclear | Low | Not reported, but before treatment. | Yes | Yes | Unclear |
|  |  | **Patient selection** | | | **Index test** | | | **Reference standard** | | | **Flow and timing** | | | |
| **Study** | **Study design** | **Patient recruitment** | **Risk of bias** | **Applicability concerns (reasons in parenthesis, see notes for abbreviations)** | **Blinding** | **Risk of bias** | **Applicability concerns** | **Blinding** | **Risk of bias** | **Applicability concerns** | **Interval between tests** | **All patients included in testing?** | **All patients included in analysis?** | **Flow and timing, risk of bias** |
| Guerra-Caceres 1979 [31] | Prospective | Unclear | Low | High (IP) | Unclear | Unclear | Unclear | Unclear | High | Low | Not reported | Yes | No | Unclear |
| Hirsowitz 1951* [32] | Prospective | Unclear | Low | High (IP) | Unclear | Unclear | Unclear | Unclear | Unclear | Low | Not reported | No | No | Unclear |
| Hoffman 1984 [10] | Prospective | Unclear | Low | High (IP) | Unclear | Low | Unclear | Unclear | High | Low | Not reported | No | No | Unclear |
| Hoffman 1986 [33] | Prospective | Unclear | Low | High (IP) | Unclear | Low | Unclear | Unclear | High | Low | Within 30 minutes | Yes | Yes | Low |
| James 1997* [34] | Prospective | Unclear | Unclear | Unclear | Unclear | Unclear | Unclear | Unclear | Unclear | Low | Not reported | Yes | No | Unclear |
| Ling 1940 [35] | Prospective | Unclear | Low | High (IP) | Unclear | Unclear | Unclear | Unclear | High | Low | Taken simultaneously. | Yes | Yes | Low |
| Ling 1948 [36] | Prospective | Unclear | Low | High (IP) | Unclear | Low | Unclear | Unclear | High | Low | Not reported | Yes | Yes | Low |
| Mehta 1984 [37] | Prospective | Unclear | Low | High (IP) | Unclear | High | Unclear | Unclear | High | Low | Not reported | Yes | Yes | Unclear |
| Ott 1938 (German)* [38] | Retrospective | Unclear | High | High (HI) | No | Unclear | Unclear | No | Unclear | Low | Not reported | No | Unclear | High |
| Rajagopal 1986 [39] | Prospective | Unclear | Low | High (IP) | Unclear | Unclear | Unclear | Unclear | High | Low | Not reported | Yes | Yes | Unclear |
| Rubin 1989 [40] | Prospective | Unclear | Low | High (IP) | Unclear | Unclear | Unclear | Unclear | Unclear | Low | Not reported | Yes | Yes | Unclear |
| Sacks 1941* [41] | Prospective | Unclear | Unclear | High (IP, HI) | Unclear | Unclear | Unclear | No | Low | Low | <2 days apart | Yes | Yes | Low |
| Schlack 1966 (Spanish) [42] | Prospective | Unclear | Low | High (IP) | Unclear | High | Unclear | Unclear | Low | Low | Within 24 hours. | Yes | Yes | Low |
| Seidenstucker 1949 (German) [43] | Retrospective | Unclear | Unclear | High (HI) | Unclear | High | Unclear | Unclear | Unclear | Low | Not reported | No | Unclear | High |
| Sekarwana 1989 [44] | Prospective | Unclear | Low | Unclear | Unclear | Low | Unclear | Unclear | High | Low | Not specific, prior to treatment. | Yes | Yes | Low |
| Seshadri 1977 [45] | Prospective | Unclear | Low | Unclear | Unclear | High | Unclear | Unclear | High | Low | Taken simultaneously. | Yes | Yes | Low |
| Shin 1994* [46] | Prospective | Unclear | Unclear | Unclear | Unclear | Unclear | Unclear | Unclear | Unclear | Low | Not reported | No | No | Unclear |
| Storti 1937 (French)* [47] | Prospective | Unclear | Low | High (IP, HI) | Unclear | Unclear | Unclear | Unclear | Unclear | Low | Taken simultaneously. | Yes | Yes | Low |
| Terminel 1973 (Spanish)* [48] | Prospective | Unclear | Low | Unclear | Unclear | Unclear | Unclear | Unclear | Unclear | Low | Not reported | No | No | Unclear |
| Vallenas 1985 [49] | Prospective | Unclear | Low | Unclear | Unclear | Unclear | Unclear | Unclear | High | Low | Not reported | Yes | No | Unclear |
| Wain 2008 [50] | Prospective | Unclear | Low | High (IP) | Unclear | Low | Unclear | Unclear | Low | Low | Within 1 day | No | Yes | High |
|  |  | **Patient selection** | | | **Index test** | | | **Reference standard** | | | **Flow and timing** | | | |
| **Study** | **Study design** | **Patient recruitment** | **Risk of bias** | **Applicability concerns (reasons in parenthesis, see notes for abbreviations)** | **Blinding** | **Risk of bias** | **Applicability concerns** | **Blinding** | **Risk of bias** | **Applicability concerns** | **Interval between tests** | **All patients included in testing?** | **All patients included in analysis?** | **Flow and timing, risk of bias** |
| West 1989* [51] | Prospective | Unclear | Low | High (IP) | Unclear | Low | Unclear | Unclear | Unclear | Low | Not reported | Yes | Yes | Unclear |
| **Note regarding the QUADAS-II tool:** | | | | | | | | | | | | | | |
| **Study design:** Was the study carried out prospectively or retrospectively (record review) [Domain 1A] Studies were categorized as "prospective" studies if the data collected for the purpose of evaluating culture diagnostic sensitivity, and studies were categorized as "retrospective" if it consisted of a record review. | | | | | | | | | | | | | | |
| **Patient recruitment:** How were the study subjects recruited? [Domain 1A] | | | | | | | | | | | | | | |
| **Patient selection risk of bias:** Could the selection of patients have introduced bias? [Domain 1A - final question] Prospective studies were considered "low" risk and retrospective studies were considered "high" risk. | | | | | | | | | | | | | | |
| **Patient selection applicability:** Is there a concern that the included patients differ from patients to whom the diagnostic test will be administered? [Domain 1B - final question] We considered studies performed in a low- to middle-income country with outpatient populations as well as inpatient populations to have a "low" risk of bias, and all others to have a high risk of bias. Studies conducted in the US, Canada, and Europe where marked HI (for high-income setting) and studies carried out in inpatient populations only were marked as IP in parenthesis. No studies were conducted on exclusively outpatient populations. | | | | | | | | | | | | | | |
| **Index test (blood culture), blinding:** Were the index test results interpreted without knowledge of the results of the reference standard? [Domain 2A] Answers can be yes, no, and unclear. | | | | | | | | | | | | | | |
| **Index test (blood culture), risk of bias:** Could the conduct or interpretation of the index test have introduced bias? [Domain 2A - final question] If the blood sample was incubated and cultured for less than 7 days, then we considered the study to have a "high risk" of bias in this domain. | | | | | | | | | | | | | | |
| **Index test (blood culture), applicability concerns:** Is there a concern that the included patients differ from patients to whom the diagnostic test will be administered? [Domain 2B - final question] We considered all studies unclear in this regard. | | | | | | | | | | | | | | |
| **Reference standard (bone marrow culture), blinding:** Were the reference test results interpreted without knowledge of the results of the index test? [Domain 3A] Answers can be yes, no, and unclear. | | | | | | | | | | | | | | |
| **Reference standard (bone marrow culture), risk of bias:** Could the conduct or interpretation of the reference standard have introduced bias? [Domain 3A - final question] Studies were considered to pose a low risk of bias if more than 1 mL of bone marrow was cultured and a high risk of bias if less than 1 mL of bone marrow was culture. | | | | | | | | | | | | | | |
| **Reference standard (bone marrow culture), applicability concerns:** Is there a concern that the reference standard does not adequately identify the target condition? [Domain 3B - final question] Likely to be low for everyone. | | | | | | | | | | | | | | |
| **Flow and timing, interval between tests:** Was there any interval between the reference standard and the index test? How long? [Domain 4A] | | | | | | | | | | | | | | |
| **Flow and timing, patients excluded from testing:** Did all patients receive the reference standard/index test? If not, did the authors state whether some groups were more likely to receive these than others? [Domain 4A] | | | | | | | | | | | | | | |
| **Flow and timing, patients excluded from analysis:** Were all patients included in the analysis? If no, who was not included? [Domain 4A] | | | | | | | | | | | | | | |
| **Flow and timing, risk of bias:** Could the patient flow have introduced bias? [Domain 4A - final question] Studies were considered to pose a low risk of bias if the sample for the cultures were taken less than 1 day apart and all patients were included in the analysis, and studies were considered to pose a high risk of bias otherwise. | | | | | | | | | | | | | | |
| **Notes on specific studies:** | | | | | | | | | | | | | | |
| **Benavente 1984:** It's not explicitly stated that it included inpatients and outpatients, but it says all patients who came to seek their services at the hospital, so we assumed it was both kinds of patients. | | | | | | | | | | | | | | |
| **Bhutta 1991:** Also high concern that patients differ from general population of patients because these were exactly the kinds that are suspected to be BC- (previous antibiotic use or long illness). | | | | | | | | | | | | | | |
| **Chaicumpa 1992:** Time between BC and BMAC culture was not stated, but table 3 indicates that antibiotic treatment may have started in the time between the two cultures. | | | | | | | | | | | | | | |
| **Chang 1982**: It is not clear what populations received the reference test (bone marrow culture) but it is clear that not all eligible patients received it. | | | | | | | | | | | | | | |
| **Dance 1991**: No clear description of patient flow or inclusion/exclusion criteria other than "30 patients with suspected enteric fever". | | | | | | | | | | | | | | |
| **Del Negro 1960:** Not sure why most patients did not receive the bone marrow cultures, but those that had bone marrow cultures were included in the analysis. | | | | | | | | | | | | | | |
| **Gasem 1995**: Bone marrow sample was only taken between 1 to 10 days of in-hospital antibiotic treatment due to staff shortages (I.e. patients had been treated for typhoid when bone marrow sample was taken) | | | | | | | | | | | | | | |
| **Guerra-Caceres 1979:** Of the 66 patients, only 60 patients completed the procedures as scheduled. No description of the other 6 patients. | | | | | | | | | | | | | | |
| **Hoffman 1984:** Time between specimen acquisition was not reported were not discussed, but one table (3) shows that there were fewer antibiotic-naive patients for the BMAC than for the BC, indicating that BMAC was done later, potentially after the beginning of treatment. | | | | | | | | | | | | | | |
| **James 1997:** 51 patients had clinical features of typhoid fever. However, only 36 patients fulfilled the inclusion criteria. | | | | | | | | | | | | | | |
| **Seidenstucker 1949:** Not all eligible patients had both the blood culture and bone marrow tests done, but the reason for that some patients had only one test done is unclear.. | | | | | | | | | | | | | | |
| **Terminel 1973:** Patients who were rose-spot culture-negative were excluded from the analysis. | | | | | | | | | | | | | | |
| **Vallenas 1985:** Patients who could not tolerate the duodenal string device were excluded from the analysis; 47% of 2-6 year olds tolerated the duodenal string device, whereas 89% of 7-13 year olds were able to tolerate it. | | | | | | | | | | | | | | |

**Table S6. Model parameter estimates using a linear meta-regression.**

|  | Among all culture positive samples | Among bone-marrow culture positive samples |
| --- | --- | --- |
| Coefficients |  |  |
| Intercept | 0.41 (0.30, 0.53), *p<*0.001 | 0.40 (0.33, 0.57), *p=*0.001 |
| Increase in sensitivity for each mL increase in volume of blood drawn. | 0.03 (0.01, 0.06), *p=*0.003 | 0.04 (0.004, 0.07), *p=*0.02 |

**Table S7. Model parameter estimates and Widely Applicable Information Criterion (WAIC) scores for models estimated with studies published after 1980.** We show the model parameter estimates as well as the goodness-of-fit measures (WAIC) for the meta-regression models of the relationship between specimen volume and blood culture sensitivity among patients with at least one culture-positive specimen and among bone-marrow culture positive patients only. For parameters that account for random effects, we also report the mean and variance hyper-parameters.

|  | **Among any culture-positive samples** | **Bone marrow positive** |
| --- | --- | --- |
| ***Model A*** |  |  |
| **Intercept parameter (*μ*)** | 0.84 (0.69, 1.03) ^a^ | 0.88 (0.67, 1.14) ^b^ |
| **WAIC** | 148.4 | 112.6 |
| ***Model B*** |  |  |
| **Slope parameter (*β*)** | 0.19 (0.16, 0.23) ^c^ | 0.18 (0.15, 0.23) ^d^ |
| **WAIC** | 196.4 | 144.6 |
| ***Model C*** |  |  |
| **Intercept parameter (*μ*)** | 0.70 (0.52, 0.90) ^e^ | 0.88 (0.67, 1.13) ^f^ |
| **Slope parameter (*β*)** | 0.04 (0.02, 0.06)* | 0.005 (0.0002, 0.02)* |
| **WAIC** | 130.3 | 83.7 |

*In Model C, which includes both an intercept and a slope, we only account for random effects in the intercept

^a^ Parameters for the lognormal distribution: Mean: -0.17 (-0.38, 0.03), SD: 8.27 (3.16, 20.83)

^b^ Parameters for the lognormal distribution: Mean: -0.13 (-0.39, 0.13), SD: 5.97 (2.07, 15.63)

^c^ Parameters for the lognormal distribution: Mean: -1.66 (-1.85, -1.46), SD: 9.64 (5.52, 26.34)

^d^ Parameters for the lognormal distribution: Mean: -1.69 (-1.93, -1.46), SD: 8.24 (2.79, 23.67)

^e^ Parameters for the lognormal distribution: Mean: -0.35 (-0.65, -0.10), SD: 6.93 (2.34, 20.70)

^f^ Parameters for the lognormal distribution: Mean: -0.12 (-0.40, 0.13), SD: 6.80 (2.27, 19.53)

**Table S8. Two-way analysis of study characteristics by publication date.**

|  | Publication Date | | |
| --- | --- | --- | --- |
|  | Before 1980 (N=8) | 1980-1990 (N=9) | After 1990 (N=8) |
| Age |  |  |  |
| *Children* | 2 | 4 | 1 |
| *Older Children and Adults* | 3 | 5 | 2 |
| *Adults* | 0 | 0 | 4 |
| *All Ages* | 1 | 1 | 1 |
| *Not reported* | 9 | 3 | 4 |
| Fisher’s exact test: *p*=0.43 (among studies that reported age group) | | | |
| Improved techniques used to culture blood |  |  |  |
| *Yes* | 3 | 7 | 5 |
| *No* | 5 | 2 | 3 |
| Chi-squared test for trend: *p*=0.31 | | | |

# Supplement S7: Supplementary Figures

**Figure S1. Sensitivity of blood culture to detect typhoid fever among bone marrow culture-confirmed typhoid patients and subgroup analysis by age.** The sensitivity of blood culture is expressed as the proportion of patients who had a positive bone marrow culture for *Salmonella* Typhi or *Salmonella* Paratyphi. The size of each marker is proportional to the number of patients in the study. We reported the midpoint volume of the blood specimen for studies that reported specimen volume as a range. Group-level and subgroup-level estimates by age are derived from random-effects models We tested for heterogeneity and age-related subgroup differences via the Q-statistic, which is assumed to have a chi-squared distribution with degrees of freedom equal to the number of studies minus 1 with non-centrality parameter equal to 0. Gasem 1995 reported sensitivity on the same patient population using specimens of two different sizes per patient, so we have taken only the results from the larger specimen in the subgroup analysis by age in order to avoid double-counting. Abbreviations: mL: milliliters, BC+: blood culture-positive, BC-: blood culture-negative, CI: confidence interval.

**
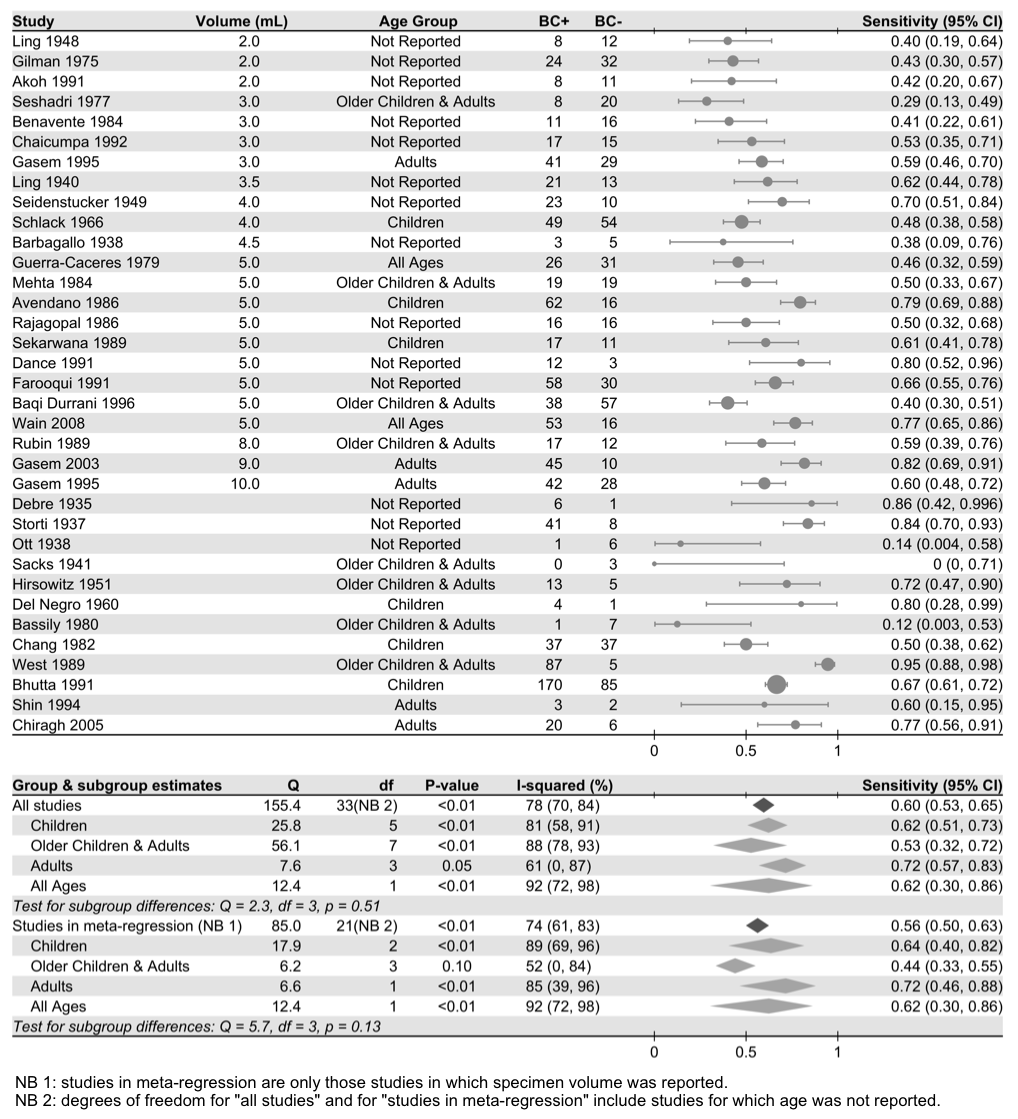
**

**Figure S2.** **Relationship between sample volume and model estimates of blood culture sensitivity among bone marrow culture-confirmed typhoid patients.** The observed blood culture sensitivity among bone-marrow positive cases is plotted in black (with corresponding 95% confidence intervals), while the mean model-predicted blood culture sensitivity is plotted in pink. The lighter pink region corresponds to the model-predicted population response for: (A) The model assumes no correlation with blood volume; (B) the model assumes sensitivity increases with increasing sample volume and is constrained to be zero for a hypothetical 0 mL sample; (C) the model assumes sensitivity increases with increasing sample volume and estimates an intercept for a hypothetical 0 mL sample. All models account for heterogeneity between studies using random effects (see Text S1). The size of the black markers corresponds to the size of the studies.


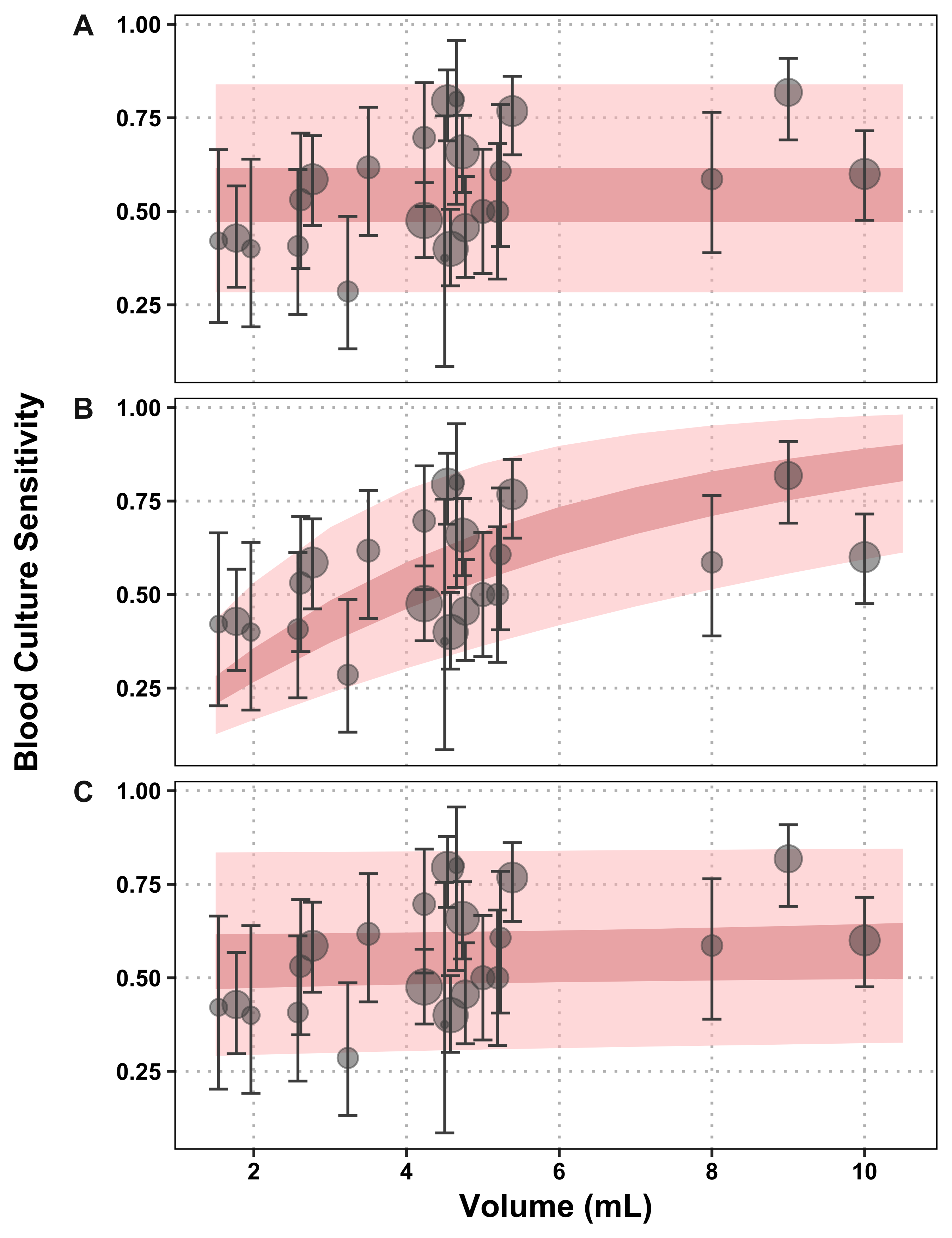


**Figure S3.** **Relationship between sample volume and estimates of blood culture sensitivity using a linear meta-regression model.** The observed blood culture sensitivity among (A) patients who were culture-positive based on any sample, and (B) bone-marrow positive cases is plotted in black (with corresponding 95% confidence intervals), while the mean model-predicted blood culture sensitivity is plotted in pink. Hoffman 1986 and Gasem 1995 reported sensitivity on the same patient population using specimens of two different sizes per patient, so we have taken only the results from the larger specimen in each study for this analysis. The lighter pink region corresponds to the model-predicted population response (see Text S1 for details and Table S6 for model parameter estimates). The size of the black markers corresponds to the size of the studies.


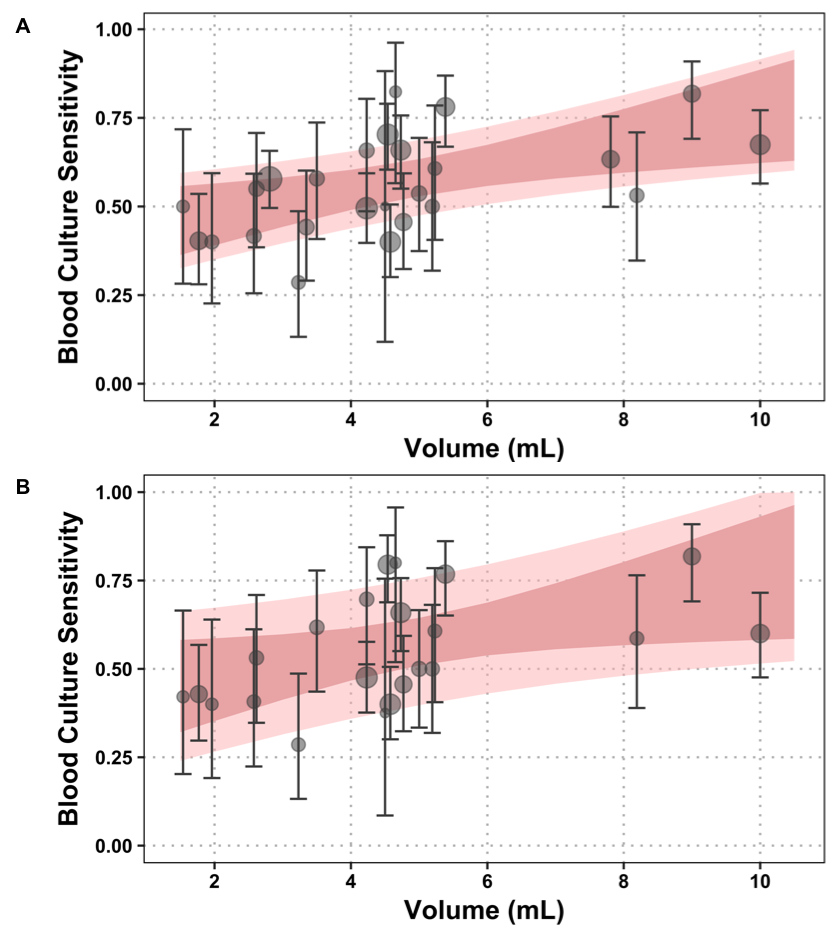


**Figure S4. Sensitivity of blood culture to detect typhoid fever among bone marrow culture-confirmed typhoid patients and subgroup analysis by decade.** The sensitivity of blood culture is expressed as the proportion of (A) patients who had a positive culture (of any site) for *Salmonella* Typhi or *Salmonella* Paratyphi and (B) as a proportion of the patients who had a positive bone marrow culture specifically (information which was available from 34 out of 40 studies). Group-level and subgroup-level estimates by age are derived from random-effects models We tested for heterogeneity and subgroup differences according to the publication date of the study via the Q-statistic, which is assumed to have a chi-squared distribution with degrees of freedom equal to the number of studies minus 1 with non-centrality parameter equal to 0. Abbreviations: CI: confidence interval.


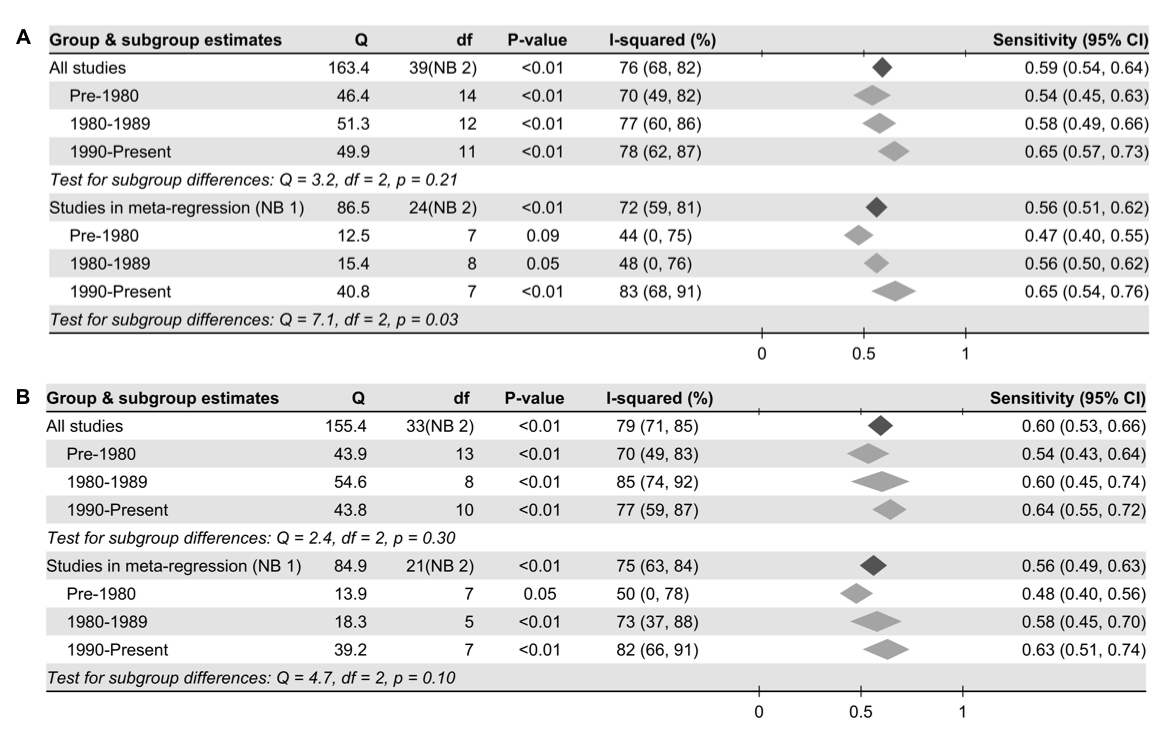


**Figure S5.** **Relationship between sample volume and model estimates of blood culture sensitivity among culture-confirmed typhoid patients in studies published after 1980.** The observed blood culture sensitivity among any culture-positive cases is plotted in black (with corresponding 95% confidence intervals), while the mean model-predicted blood culture sensitivity is plotted in pink. The lighter pink region corresponds to the model-predicted population response for: (A) The model assumes no correlation with blood volume; (B) the model assumes sensitivity increases with increasing sample volume and is constrained to be zero for a hypothetical 0 mL sample; (C) the model assumes sensitivity increases with increasing sample volume and estimates an intercept for a hypothetical 0 mL sample. All models account for heterogeneity between studies using random effects (see Text S1). The size of the black markers corresponds to the size of the studies.


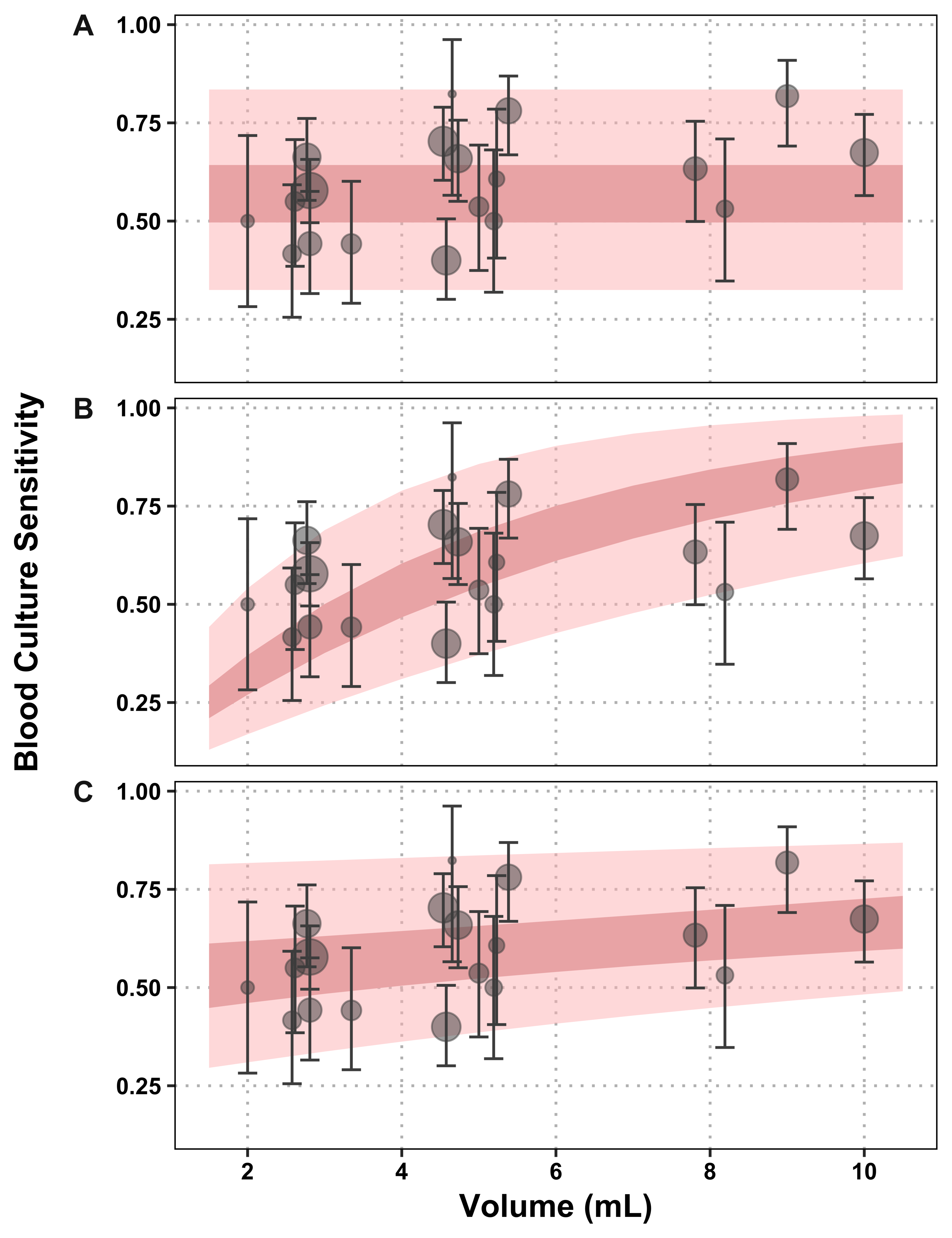


**Figure S6.** **Relationship between sample volume and model estimates of blood culture sensitivity among bone marrow culture-confirmed typhoid patients in studies published after 1980.** The observed blood culture sensitivity among bone-marrow positive cases is plotted in black (with corresponding 95% confidence intervals), while the mean model-predicted blood culture sensitivity is plotted in pink. The lighter pink region corresponds to the model-predicted population response for: (A) The model assumes no correlation with blood volume; (B) the model assumes sensitivity increases with increasing sample volume and is constrained to be zero for a hypothetical 0 mL sample; (C) the model assumes sensitivity increases with increasing sample volume and estimates an intercept for a hypothetical 0 mL sample. All models account for heterogeneity between studies using random effects (see Text S1). The size of the black markers corresponds to the size of the studies.


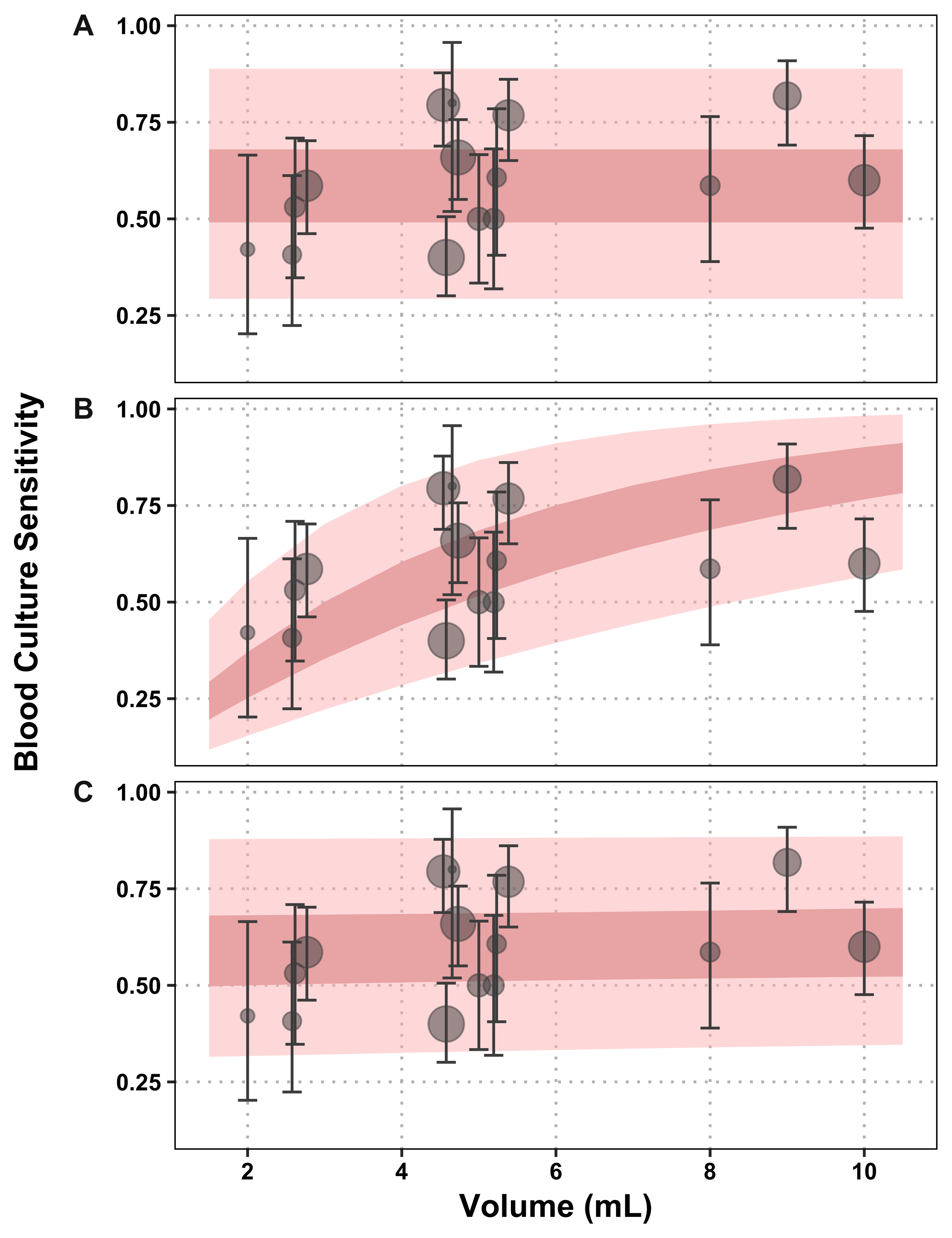


**Figure S7.** **Relationship between sample volume and model estimates of blood culture sensitivity among bone marrow culture-confirmed typhoid patients only in those studies that reported blood culture sensitivity data on both all culture-positive patients as well as on bone marrow culture-positive patients.** The observed blood culture sensitivity among all culture-positive cases in the studies used for the analysis is plotted in black (with corresponding 95% confidence intervals), the studies excluded for this analysis were plotted in red (those that did not contain information on blood culture sensitivity among all culture-confirmed cases), while the mean model-predicted blood culture sensitivity is plotted in the pink bands. The lighter pink region corresponds to the model-predicted population response for: (A) the model assumes no correlation with blood volume; (B) the model assumes sensitivity increases with increasing sample volume and is constrained to be zero for a hypothetical 0 mL sample; (C) the model assumes sensitivity increases with increasing sample volume and estimates an intercept for a hypothetical 0 mL sample. All models account for heterogeneity between studies using random effects (see Text S1). The size of the black and red markers corresponds to the size of the studies.

**
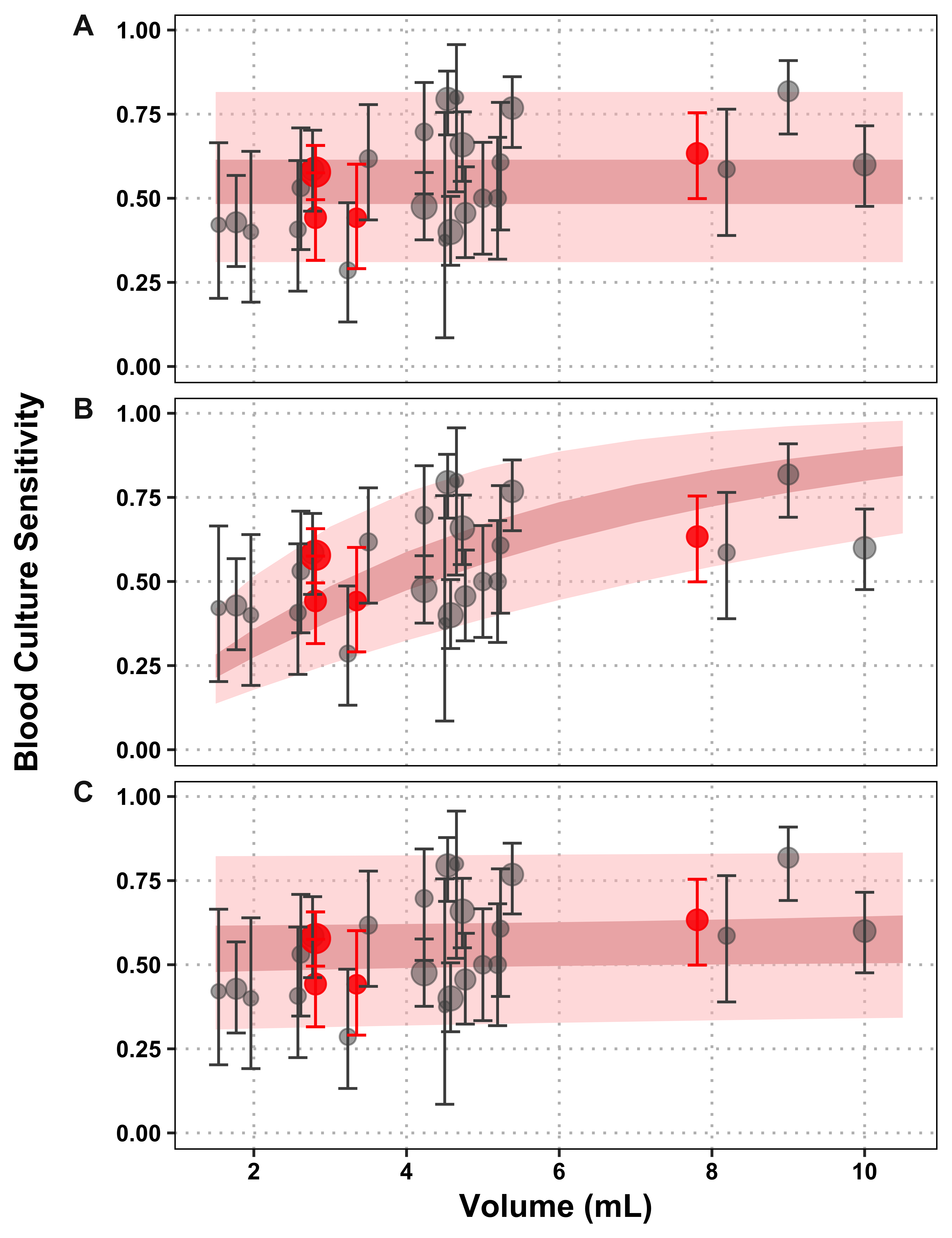
**

**Figure S8. Correlation between blood culture sensitivity as measured using two different reference standards.** Blood culture sensitivity can be measured using all culture-confirmed patients as the denominator (x-axis) or only bone marrow culture-confirmed cases as the denominator (y-axis). Studies that lie on the dashed 45-degree line had sensitivity estimates that were identical irrespective of the reference standard. The size of the orange markers corresponds to the volume of blood culture specimen.


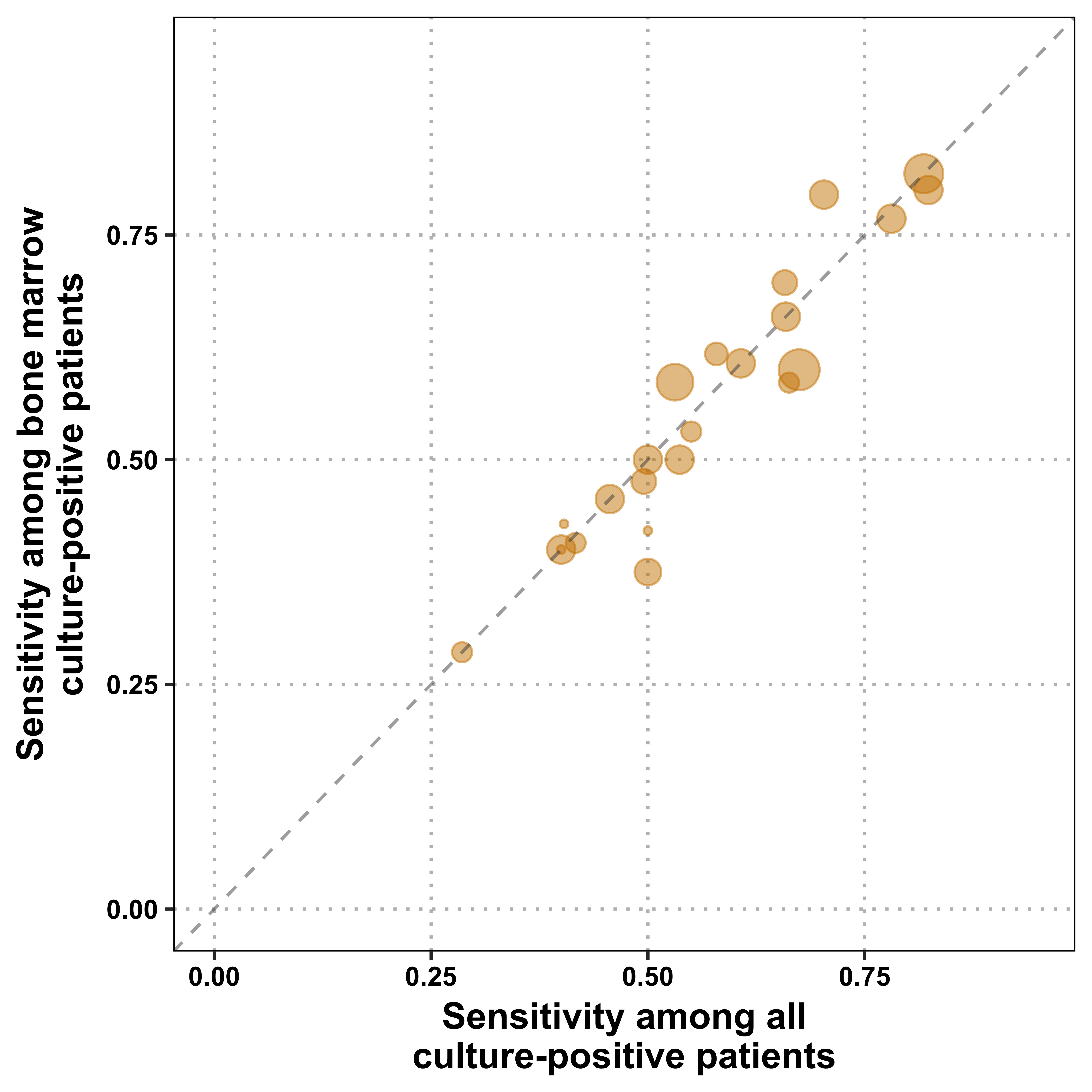


# Supplement S8. Supplement References

[1] Seshadri V, Natarajan K, Sundaravelu T, Jepegnanum J, Jeyaraj SD, Gnanavendan. Efficacy of bone marrow culture in enteric fever. J Assoc Physicians India 1977;25:561–4.

[2] Schwarzer G. meta: An R package for meta-analysis. R News 2007;7:40–5.

[3] Viechtbauer W. Conducting Meta-Analyses in R with the metafor Package. J Stat Softw 2010;36. doi:10.18637/jss.v036.i03.

[4] Bates D, Maechler M, Bolker B, Walker S. Fitting Linear Mixed-Effects Models Using lme4. J Stat Softw 2015;67:1–48. doi:10.18637/jss.v067.i01.

[5] Hothorn AZ and T. Diagnostic Checking in Regression Relationships. R News 2002;2:7–10. doi:10.13140/RG.2.1.3265.7761.

[6] Plummer M. JAGS: A program for analysis of Bayesian graphical models using Gibbs sampling. Proc 3rd Int Work Distrib Stat Comput 2003:1–10. doi:ISSN 1609-395X.

[7] Plummer M. rjags: Bayesian Graphical Models using MCMC 2016.

[8] Wickham H. ggplot2: Elegant Graphics for Data Analysis. Springer-Verlag New York; 2009.

[9] World Health Organization. Making Choices in Health: WHO Guide to Cost-Effectiveness Analysis. Geneva, Switzerland: World Health Organization; 2002.

[10] Hoffman SL, Punjabi NH, Rockhill RC, Sutomo A, Rivai AR, Pulungsih SP, et al. Duodenal String-Capsule Culture Compared with Bone-Marrow, Blood, and Rectal-Swab Cultures for Diagnosing Typhoid and Para-Typhoid Fever. J Infect Dis 1984;149:157–61.

[11] World Health Organization. Background document: The diagnosis, treatment and prevention of typhoid fever. 2003.

[12] Crump JA, Kirk MD. Estimating the Burden of Febrile Illnesses. PLoS Negl Trop Dis 2015;9 (12) (no. doi:http://dx.doi.org/10.1371/journal.pntd.0004040.

[13] Akoh JA. Relative sensitivity of blood and bone marrow cultures in typhoid fever. Trop Doct 1991;21:174–6.

[14] Avendano A, Herrera P, Horwitz I, Duarte E, Prenzel I, Lanata C, et al. Duodenal string cultures: practicality and sensitivity for diagnosing enteric fever in children. J Infect Dis 1986;153:359–62.

[15] Durrani AB, Rab SM. Changing spectrum of typhoid. J Pak Med Assoc 1996;46:50–2.

[16] Barbagallo G. Sterno-medullar culture in infective diseases. Policlinico 1938;45:230–43.

[17] Bassily S, Farid Z, Watten RH. Bone marrow culture in chronic salmonellosis. Trans R Soc Trop Med Hyg 1980;74:829–30.

[18] Benavente L, Gotuzzo E, Guerra J, Grados O, Guerra H. Diagnosis of Salmonella Typhi by culture of duodenal string capsule. N Engl J Med 1981;304:54.

[19] Benavente L, Gotuzzo E, Guerra J, Grados O, Guerra H, Bravo N. Diagnosis of typhoid fever using a string capsule device. Trans R Soc Trop Med Hyg 1984;78:404–6. doi:10.1016/0035-9203(84)90134-2.

[20] Bhutta ZA. Bone marrow examination in prolonged fever. J Pediatr 1991;119:840–1.

[21] Chaicumpa W, Ruangkunaporn Y, Burr D, Chongsa-Nguan M, Echeverria P. Diagnosis of typhoid fever by detection of Salmonella typhi antigen in urine. J Clin Microbiol 1992;30:2513–5.

[22] Chang JE, Hernández H, Yi A, Chea E, Chaparro E, Matos E, et al. [Hemoculture and bone marrow culture in children with typhoid fever]. Bol Med Hosp Infant Mex 1982;39:614–6.

[23] Chiragh S, ur Rehman S. Blood and bone marrow culture sensitivity in clinically suspected enteric fever. J Postgrad Med Inst 2005;19:313–6.

[24] Dance D, Richens JE, Ho M, Acharya G, Pokhrel B, Tuladhar NR. Blood and bone marrow cultures in enteric fever. J Clin Pathol 1991;44:1038. doi:10.1136/jcp.44.12.1038-a.

[25] Debre R, Lamy M, Bonnet H, Broca R. La medulloculture. Bull Mémoires La Soc Medicale Des Hop Paris 1935:1723–7.

[26] Del Negro G. Clinical Aspects of Typhoid Fever in Children; Analysis of 60 Cases. Rev do Hosp das Clin Fac Med da Univ Sao Paulo 1960;15:394–404.

[27] Farooqui BJ, Khurshid M, Ashfaq MK, Khan MA. Comparative yield of Salmonella typhi from blood and bone marrow cultures in patients with fever of unknown origin. J Clin Pathol 1991;44:258–9. doi:10.1136/jcp.44.3.258.

[28] Gasem MH, Dolmans WM, Isbandrio BB, Wahyono H, Keuter M, Djokomoeljanto R, et al. Culture of Salmonella typhi and Salmonella paratyphi from blood and bone marrow in suspected typhoid fever. Trop Geogr Med 1995;47:164–7.

[29] Gasem MH, Keuter M, Dolmans WM V, Van Der Ven-Jongekrijg J, Djokomoeljanto R, Van Der Meer JWM. Persistence of Salmonellae in blood and bone marrow: randomized controlled trial comparing ciprofloxacin and chloramphenicol treatments against enteric fever. Antimicrob Agents Chemother 2003;47:1727–31.

[30] Gilman RH, Terminel M, Levine MM, Hernandez-Mendoza P, Hornick RB. Relative efficacy of blood, urine, rectal swab, bone-marrow, and rose-spot cultures for recovery of Salmonella typhi in typhoid fever. Lancet 1975;1:1211–3.

[31] Guerra-Caceres JG, Gotuzzo-Herencia E, Crosby-Dagnino E, Miro-Quesada M, Carrillo-Parodi C. Diagnostic value of bone marrow culture in typhoid fever. Trans R Soc Trop Med Hyg 1979;73:680–3. doi:10.1016/0035-9203(79)90020-8.

[32] Hirsowitz L, Cassel R. Sternal marrow cultures in typhoid fever. Br Med J 1951;1:862–3.

[33] Hoffman SL, Edman DC, Punjabi NH, Lesmana M, Cholid A, Sundah S, et al. Bone marrow aspirate culture superior to streptokinase clot culture and 8 ml 1:10 blood-to-broth ratio blood culture for diagnosis of typhoid fever. Am J Trop Med Hyg 1986;35:836–9.

[34] James J, Dutta TK, Jayanthi S. Correlation of clinical and hematologic profiles with bone marrow responses in typhoid fever. Am J Trop Med Hyg 1997;57:313–6.

[35] Ling CC, Taur SS, Hsueh PC, Yang SY. Medulloculture in the Diagnosis of Typhoid and Paratyphoid Fevers. An Analysis of 38 Cases. Chin Med J (Engl) 1940;57:11–26.

[36] Ling C, Liu J, Chen T. A comparative study of bile, marrow, blood, stool, urine cultures and Widal reaction in typhoid and paratyphoid fevers an analysis of 31 cases. Chin Med J (Engl) 1948;66:66–78.

[37] Mehta GC, Joshi BU, Tilak SS. Evaluation of the bone marrow culture versus the blood culture for laboratory diagnosis of enteric fever. Indian J Med Sci 1984;38:21–2.

[38] Ott A. The Importance of Bone Marrow Culture for Isolation of Typhoid and Paratyphoid Bacilli. Klin Wochenschr 1938;17:1475–6.

[39] Rajagopal V, Kerur DK, Bhargava SK, Prabhu T. Bone marrow culture in the diagnosis of enteric fever. Indian J Pathol Microbiol 1986;29:15–9.

[40] Rubin FA, McWhirter PD, Punjabi NH, Lane E, Sudarmono P, Pulungsih SP, et al. Use of a DNA probe to detect Salmonella typhi in the blood of patients with typhoid fever. J Clin Microbiol 1989;27:1112–4.

[41] Sacks MS, Hachiel FW. A note on the bacteriologic culture of bone marrow in typhoid fever. J Lab Clin Med 1941;26:1024–9.

[42] Schlack L, Pino M, Wiederhold A. Myeloculture in the diagnosis of typhoid and paratyphoid fevers. Comparative analysis of 135 cases upon hospital admission. [Spanish]. Rev Chil Pediatr 1966;37:213–20.

[43] Seidenstucker H. The Importance of Blood and Bone Marrow Culture for Diagnosis of the Enteric Group of Infections. Dtsch Medizinische Wochenschrift 1949;74:1434–6. doi:http://dx.doi.org/10.1055/s-0028-1121381.

[44] Sekarwana N, Garna H, Azhali MS. Results of Salmonella typhi culture in patients with suspected typhoid fever, treated in the Department of Child Health Medical School, Padjadjaran University, Hasan Sadikin General Hospital Bandung. Paediatr Indones 1989;29:105–11.

[45] Seshadri V, Natarajan K, Sundaravelu T, Jepegnanum J, Jeyaraj SD, Gnanavendan. Efficacy of bone marrow culture in enteric fever. J Assoc Physicians India 1977;25:561–4.

[46] Shin BM, Paik IK, Cho HI. Bone marrow pathology of culture proven typhoid fever. J Korean Med Sci 1994;9:57–63.

[47] Storti E, Filippi C. Etude morphologique et bacteriologique de la moelle osseuse dans la fievre typhoide. Le Sang Biol Pathol 1937:440–4.

[48] Terminel VM, Mendoza HP, Bahena AI. New technique for biopsy and culture of rose spots in the diagnosis of typhoid fever. Rev Latinoam Microbiol 1973;15:69–70.

[49] Vallenas C, Hernandez H, Kay B, Black R, Gotuzzo E. Efficacy of bone marrow, blood, stool and duodenal contents cultures for bacteriologic confirmation of typhoid fever in children. Pediatr Infect Dis J 1985;4:496–8.

[50] Wain J, Diep TS, Bay PVB, Walsh AL, Vinh H, Duong NM, et al. Specimens and culture media for the laboratory diagnosis of typhoid fever. J Infect Dev Ctries 2008;2:469–74. doi:http://dx.doi.org/10.3855/jidc.164.

[51] West B, Richens JE, Howard PF. Evaluation in Papua New Guinea of a urine coagglutination test and a widal slide agglutination test for rapid diagnosis of typhoid fever. Trans R Soc Trop Med Hyg 1989;83:715–7. doi:10.1016/0035-9203(89)90407-0.

[52] Crump JA, Sjölund-Karlsson M, Gordon MA, Parry CM. Epidemiology, clinical presentation, laboratory diagnosis, antimicrobial resistance, and antimicrobial management of invasive Salmonella infections. Clin Microbiol Rev 2015;28:901–37. doi:10.1128/CMR.00002-15.

[53] Watson KC. Laboratory and clinical investigation of recovery of Salmonella typhi from blood. J Clin Microbiol 1978;7:122–6.

[54] Escamilla J, Santiago LT, Sangalang RP, Ranoa CP, Cross JH. Comparative study of three blood culture systems for isolation of enteric fever Salmonella. Southeast Asian J Trop Med Public Health 1984;15:161–6.

[55] Escamilla J, Santiago LT, Uylangco C V, Cross JH. Evaluation of sodium polyanethanol sulfonate as a blood culture additive for recovery of Salmonella typhi and Salmonella paratyphi A. J Clin Microbiol 1983;18:380–3.
